# Physician-revised AI-generated drafts are associated with higher ratings of written explanations in end-of-life care in the intensive care unit: a scenario-based single-center cross-sectional study

**DOI:** 10.1186/s40560-026-00930-2

**Published:** 2026-08-24

**Authors:** Atsushi Kokita, Junpei Haruna, Yuya Goto, Soichi Tanaka, Satoshi Sato, Kenichiro Kikuchi, Masayuki Akatsuka, Ryu Azumaguchi, Ikuto Otsuki, Michiaki Yamakage, Satoshi Kazuma

**Affiliations:** 1 Department of Anesthesiology, Sapporo Medical University School of Medicine, Sapporo, Hokkaido Japan; 2 Department of Intensive Care Medicine, Sapporo Medical University School of Medicine, Sapporo, Hokkaido Japan; 3 Department of Anesthesiology, Otaru General Hospital, Otaru, Hokkaido Japan

**Keywords:** End-of-life care, Artificial intelligence, Large language model, Medical validity, Empathy

## Abstract

**Background:**

In end-of-life care (EOL) in the intensive care unit (ICU), intensivists are expected to provide medically appropriate and empathetic communication to support shared decision-making with patients and their families. Large language models (LLMs) have shown potential to generate medical responses that are perceived as informative and empathetic, and collaborative workflows involving physician review of LLM-generated drafts have been evaluated, particularly for ambulatory patient portal messaging. However, it remains unclear whether this collaborative approach is associated with higher ratings of communication quality in ICU EOL care. In this study, we used clinical scenarios simulating ICU EOL situations to compare the quality of written explanations generated by physicians alone, an LLM-based chatbot alone, and physicians who revised LLM-generated drafts.

**Methods:**

In this preliminary, scenario-based study, a total of 45 participants were enrolled, including healthcare professionals and nonhealthcare professionals. Three clinical scenarios simulating ICU EOL care and questions from patients or family members were prepared. For each question, three types of responses were prepared: physician-only, AI-only, and physician–AI collaborative responses. The three prespecified primary outcomes were medical validity and empathy rated by healthcare professionals from diverse specialties and clinical settings, and empathy rated by nonhealthcare professionals, using 5-point Likert scales.

**Results:**

In the primary analyses, physician–AI collaborative responses received significantly higher ratings than both physician-only and AI-only responses across all three prespecified primary outcomes. The observed differences between physician–AI collaborative responses and both other response groups were more pronounced for empathy than for medical validity. Physician-only and AI-only responses did not differ significantly for any primary outcome in the primary analyses. Sensitivity and exploratory analyses supported the same overall pattern of higher ratings for physician–AI collaborative responses. By contrast, comparisons between physician-only and AI-only responses varied across analytic approaches.

**Conclusions:**

In this scenario-based study of written explanations in ICU EOL care, physician revision of LLM-generated drafts was associated with higher ratings, particularly for empathy. However, the clinical importance of these differences remains uncertain, and the contribution of the LLM-generated draft could not be distinguished from physician revision or the two-stage drafting-and-revision process. Further multicenter, process-matched prospective studies are warranted.

**Supplementary Information:**

The online version contains supplementary material available at 10.1186/s40560-026-00930-2.

## Background

End-of-life (EOL) decisions in intensive care units (ICUs) often involve withholding or withdrawing life-sustaining treatment [1]. Shared decision-making is therefore recommended to align treatment decisions with medical evidence and the patient’s values, goals, and preferences [2]. High-quality communication is vital in supporting surrogate decision-makers and reducing psychological distress among family members and bereaved relatives [3, 4]. Medically accurate and clinically appropriate information is essential for patients and their families to understand the patient’s condition, prognosis, and treatment options. At the same time, empathetic communication helps build trust between clinicians and families, provides emotional support, and facilitates the expression of patients’ and families’ concerns and values. These informational and relational elements complement each other and jointly support informed, patient-centered shared decision-making [2–5]. However, communication skills among clinicians vary, and access to formal training remains limited [6, 7]. Consequently, families may receive inadequate explanations and experience discrepancies in prognostic understanding [8].

Large language models (LLMs), a type of artificial intelligence (AI) designed to process and generate text, have recently been explored as tools to support medical communication [9]. Previous studies in general clinical settings have shown that LLMs can generate explanations that are perceived as empathetic and high quality [10, 11]. However, LLMs may also generate inaccurate or overly generalized information and may not adequately account for individual clinical circumstances, underscoring the need for physician review [12]. Human-in-the-loop workflows, in which physicians review and edit LLM-generated drafts, have also been implemented for patient portal messaging and evaluated in terms of implementation, physician workload, and response-related time [13, 14]. However, these collaborative workflows have focused primarily on patient portal messages in ambulatory care settings. It remains unclear whether physician revision of LLM-generated drafts is associated with higher ratings of communication quality in ICU EOL care.

This study aimed to compare written explanations produced by physicians alone, an LLM-based chatbot alone, or through physician revision of LLM-generated drafts in simulated ICU EOL scenarios in terms of two complementary dimensions of communication quality: medical validity and empathy. Medical validity reflected the accuracy and clinical appropriateness of the information provided, whereas empathy reflected responsiveness to the emotional needs, concerns, and values of patients and their families. We hypothesized that physician–AI collaborative responses would be rated higher than either approach alone in both medical validity and empathy.

## Methods

### Study design and setting

This was a single-center, scenario-based, cross-sectional study conducted at the Department of Intensive Care Medicine, Sapporo Medical University School of Medicine, Japan, between September 1 and November 30, 2025. This study was approved by the Ethics Committee of Sapporo Medical University (approval number: 7-1-14; June 11, 2025), and written informed consent was obtained from all participants. All collected data were anonymized and stored under password protection by the principal investigator. This study was conducted and reported in accordance with the Strengthening the Reporting of Observational Studies in Epidemiology (STROBE) statement [15].

### Participants

Participants were recruited via a study notice posted on the website of the Department of Intensive Care Medicine, Sapporo Medical University School of Medicine, and included healthcare professionals (physicians and nurses) and nonhealthcare professionals. The inclusion criteria for healthcare professionals were physicians or nurses who were not affiliated with the ICU at our hospital. Healthcare professionals were recruited across diverse specialties and clinical settings rather than specifically targeting ICU specialists. For nonhealthcare professionals, the inclusion criteria were adults (aged ≥ 18 years) with an education level of high school or above to ensure adequate comprehension of the clinical scenarios. A total of 45 participants were enrolled, comprising 15 physicians, 15 nurses, and 15 nonhealthcare professionals.

### Variables

Three prespecified primary outcomes were defined according to the combination of rating domain and rater group: (1) medical validity rated by healthcare professionals, (2) empathy rated by healthcare professionals, and (3) empathy rated by nonhealthcare professionals. For each primary outcome, scores were compared across the physician-only, AI-only, and physician–AI collaborative response groups. The secondary outcome was the association between medical validity and empathy rated by healthcare professionals. Healthcare professionals rated medical validity and empathy, whereas nonhealthcare professionals rated empathy only, using a 5-point Likert scale.

#### Medical validity

The 5-point medical validity scale was developed by the investigators specifically for this study and was not derived from a previously validated instrument. The scale was designed to assess the accuracy, clinical appropriateness, and completeness of the information provided, as well as its potential to cause misunderstanding or harm. Medical validity was rated as follows: (1) (very poor), indicating that the response contained incorrect or unfounded medical information that could cause misunderstanding or harm to the patient; (2) (poor), indicating that the response lacked sufficient medical basis and omitted important information or included inappropriate explanations; (3) (acceptable), indicating that the response contained some inaccurate or insufficient information but maintained overall medical validity; (4) (good), indicating that the response was generally appropriate and acceptable for clinical practice, with most necessary information adequately conveyed; and (5) (very good), indicating that the response was scientifically well founded, consistent with current guidelines and evidence, and required no important additional information.

#### Empathy

Empathy was rated as follows: 1 indicated not empathetic, 2 indicated slightly empathetic, 3 indicated moderately empathetic, 4 indicated empathetic, and 5 indicated very empathetic. To guide the evaluation of empathy, participants were provided in advance with a Japanese translation of the Consultation and Relational Empathy (CARE) Measure [16]. Because the original CARE Measure was developed for face-to-face clinical consultations, it was not used as a formal scoring instrument in this study; selected concepts were adapted only as a reference framework for evaluating written communication.

### Data collection

Three intensivists at our institution developed three clinical scenarios simulating ICU EOL care. The scenarios covered three representative life-threatening conditions encountered in the ICU: (1) septic shock requiring veno-arterial extracorporeal membrane oxygenation, (2) severe respiratory failure in a patient with malignancy, and (3) postcardiac arrest syndrome. For each scenario, an explanation intended for the patient or family members was prepared, including the patient’s background, clinical course, treatment, and prognosis. In addition, each scenario included five questions posed by the patient or family members to the physician, yielding a total of 15 questions across all scenarios. For each question, three types of responses were prepared: physician-only, AI-only, and physician–AI collaborative responses. Participants read all scenarios and corresponding responses, and then assessed the quality of the response texts.

Responses were generated by the following three groups. The same question-specific instructions regarding the required clinical information and empathetic communication were applied to the physician-only and physician–AI collaborative groups. These instructions required clear and empathetic explanations addressing the specified clinical information, including, as applicable, the patient’s condition, treatment rationale, potential complications, prognosis, and plan of care. The AI-only group prompt likewise emphasized medical appropriateness and empathy.

In this study, “AI” refers to ChatGPT, an LLM-based chatbot. “LLM” is used when referring to the underlying model or technology, whereas “AI” is retained in the group labels for clarity and consistency.

1. Physician-only group

Intensivists who were not involved in scenario development independently generated the responses without the assistance of LLMs. For each question, they followed the same question-specific instructions regarding the required clinical information and empathetic communication as those applied to the physician–AI collaborative group.

2. AI-only group

The clinical scenarios were entered into ChatGPT using a predefined prompt to generate medically valid and empathetic explanations for patients or families. The generated responses were used in their original form, without any modification, including correction of typographical errors.

3. Physician–AI collaborative group

Different intensivists at our institution, who were not involved in scenario development and were distinct from those assigned to the physician-only and AI-only groups, generated the physician–AI collaborative responses using ChatGPT. Specifically, the clinical scenarios were entered into ChatGPT using the same predefined prompt as that used in the AI-only group. The generated drafts were then revised by the physicians in accordance with the same question-specific instructions regarding the required clinical information and empathetic communication as those provided to the physician-only group.

Responses were generated by seven intensivists. All response authors had more than 10 years of clinical experience and were board-certified in intensive care medicine in Japan. For each scenario, physician-only and physician–AI collaborative responses were authored by different intensivists, whereas all AI-only responses were generated by ChatGPT after a single intensivist entered the scenarios and a predefined prompt. In the AI-only and physician–AI collaborative groups, only a single prompt entry was permitted for each question, and no additional instructions beyond the predefined text were allowed. The clinical scenarios and the instructions provided for each question are presented in Additional File 1. Additional File 2 contains the same questions and instructions together with the complete physician-only, AI-only, and physician–AI collaborative responses. The predefined prompt entered into ChatGPT is provided in Additional File 3.

The LLM used in this study was GPT-5.0 (ChatGPT Plus; OpenAI). All LLM-generated responses were produced between August 1 and August 15, 2025, using the web-based ChatGPT user interface. No additional system-level instructions or custom instructions were specified by the investigators other than a predefined prompt provided in Additional File 3. Because the web-based interface was used, generation parameters such as temperature, top-p, and random seed could not be manually controlled. The prompt and all generated responses were in Japanese, and no translation process was involved. For each question, only one response was generated, and variability across repeated generations was not assessed. To minimize potential bias from prior conversation history, we used a newly created account and the Temporary Chat feature for all interactions.

### Bias

For each scenario, the allocation of physicians to the physician-only and physician–AI collaborative groups was randomized by a research collaborator who was not involved in response generation or evaluation. This process was performed using sequentially numbered, opaque envelopes to ensure allocation concealment. For each of the 15 questions, every participant evaluated all three corresponding responses: one physician-only response, one AI-only response, and one physician–AI collaborative response. Each participant evaluated 45 response texts; healthcare professionals provided 90 ratings across two domains, whereas nonhealthcare professionals provided 45 empathy ratings. The three responses for each question were presented together, but their order was randomized using computer-generated random numbers. The questions were presented in a predefined sequence within each scenario to preserve the clinical and narrative continuity of the scenario; therefore, question order was not randomized. Participants were informed that the study compared different types of written responses; however, they were blinded to whether each response belonged to the physician-only, AI-only, or physician–AI collaborative group.

### Study size

Because this was a preliminary, scenario-based study and there was limited prior information regarding the expected effect size, no a priori sample size calculation based on statistical power was performed. Based on feasibility, we planned to recruit 15 participants each in the physician, nurse, and nonhealthcare professional groups.

### Statistical analysis

For each of the three prespecified primary outcomes, the three response groups were compared using the Friedman test at the participant level as the primary analysis. Linear mixed-effects models were fitted to rating-level data as sensitivity analyses, and generalized linear mixed-effects models examining the probability of a score ≥ 4 were fitted as exploratory analyses. The secondary outcome, the association between medical validity and empathy rated by healthcare professionals, was analyzed separately using a linear mixed-effects model.

For the primary analysis, scores for the 15 responses in each group (five questions across three scenarios) were averaged for each participant, and the resulting participant-level mean scores were compared across groups using the Friedman test. Kendall’s W was calculated as an effect size [17], with 95% confidence intervals (CIs) estimated using 10,000 participant-level bootstrap samples that preserved the three matched group scores within each participant. To control the family-wise error rate across the three prespecified primary outcomes, a Bonferroni-adjusted significance threshold of 0.0167 (0.05/3) was used for the Friedman tests. For outcomes with a significant difference in the Friedman test, post hoc pairwise comparisons were performed using Dunn’s multiple comparison test [18], with a two-sided *P* < 0.05 considered statistically significant.

For the sensitivity analyses of the same three primary outcomes, linear mixed-effects models were used to account for repeated ratings across questions. The models included response group as a fixed effect and random intercepts for participant and question. Because each question was linked to a specific clinical scenario, scenario-level variation was represented by the question-level random intercept. Response author was not included as a random effect because the number of authors was small and authors were not fully crossed across response groups. Parameter estimation was performed using restricted maximum likelihood, and Satterthwaite’s method was used to approximate the degrees of freedom for fixed effects [19]. Effect estimates with 95% CIs were reported.

For the exploratory analyses, scores ≥ 4 were coded as 1 and scores ≤ 3 as 0, and generalized linear mixed-effects models were used to compare the probability of receiving a score ≥ 4 across the response groups. Response group was included as a fixed effect, with random intercepts for participant and question. Odds ratios (ORs) with 95% CIs were reported.

For the secondary outcome, the association between medical validity and empathy rated by healthcare professionals was analyzed using a linear mixed-effects model. Medical validity was modeled as the dependent variable, with empathy, response group, and their interaction included as fixed effects, and participants and questions as random intercepts; group-specific *β* coefficients with 95% CIs were reported.

In a post hoc analysis, inter-rater reliability was evaluated separately for each primary outcome across the 45 response texts. Single-measure intraclass correlation coefficients (ICCs) with 95% CIs were calculated using a two-way random-effects model for absolute agreement.

In a separate post hoc analysis, response length was defined as Japanese character count excluding spaces and line breaks. Character counts were compared across matched groups using the Friedman test, followed by Dunn’s pairwise comparisons after a significant overall test. The primary-outcome linear mixed-effects models were also refitted with mean-centered character count per 100 characters as a fixed effect.

Unless otherwise specified, two-sided *P* < 0.05 was considered statistically significant. Participant-level mean scores are summarized as medians with interquartile ranges (IQRs). Generalized linear mixed models, linear mixed-effects models, and ICC analyses were fitted using R version 4.3.1 (The R Foundation for Statistical Computing, Vienna, Austria), whereas all other analyses were performed using GraphPad Prism 10 (GraphPad Software, Boston, MA, USA, version 10.6.1 [892]). All analyses were performed using complete-case data; because there were no missing data, no imputation was performed.

## Results

**Fig. 1:**
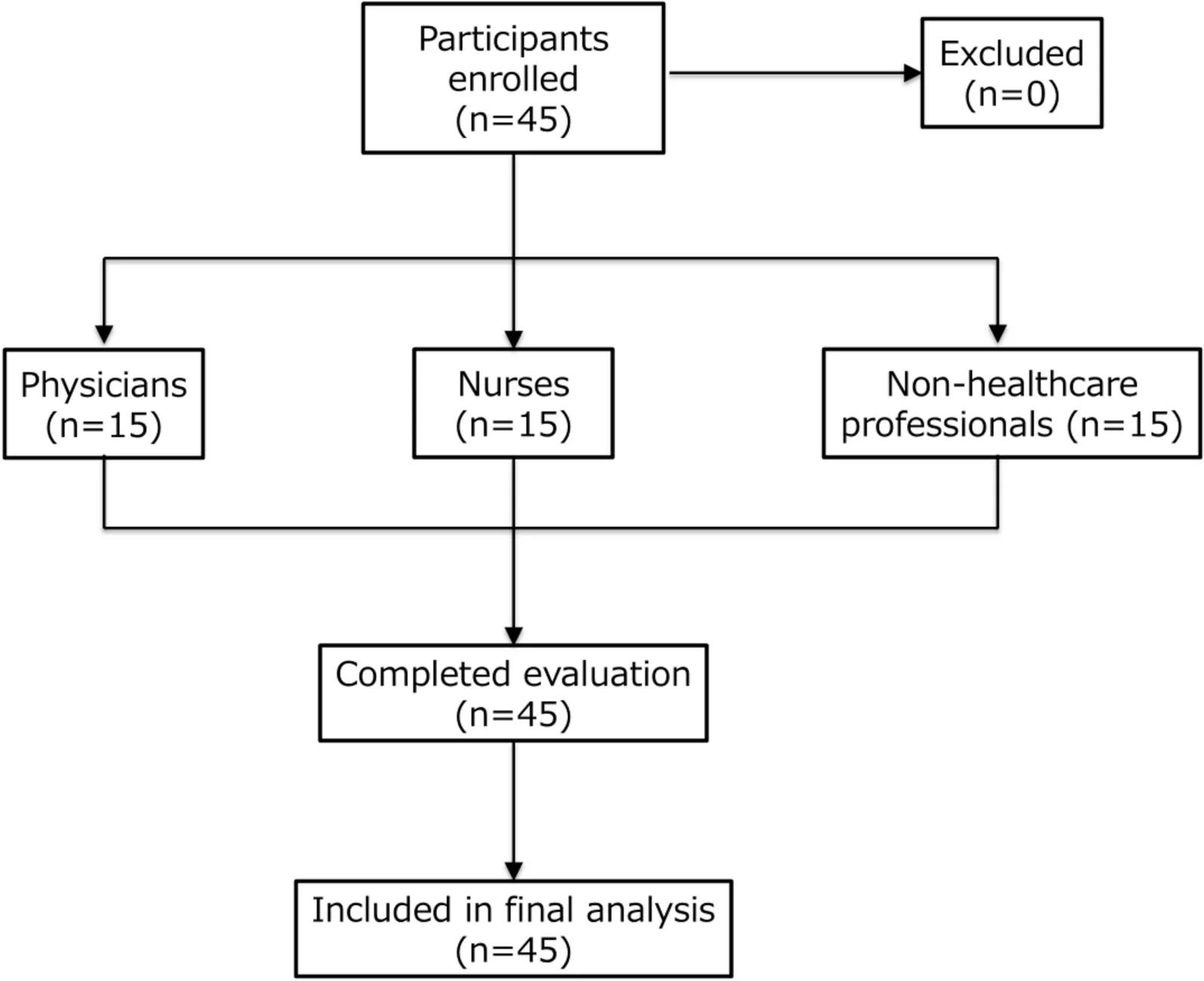
Participant flow

Between September 1 and November 30, 2025, 45 participants were enrolled, comprising 15 physicians, 15 nurses, and 15 nonhealthcare professionals. All enrolled participants evaluated all responses from each group across the three scenarios; there were no withdrawals or missing data (Fig. 1).

**Fig. 2:**
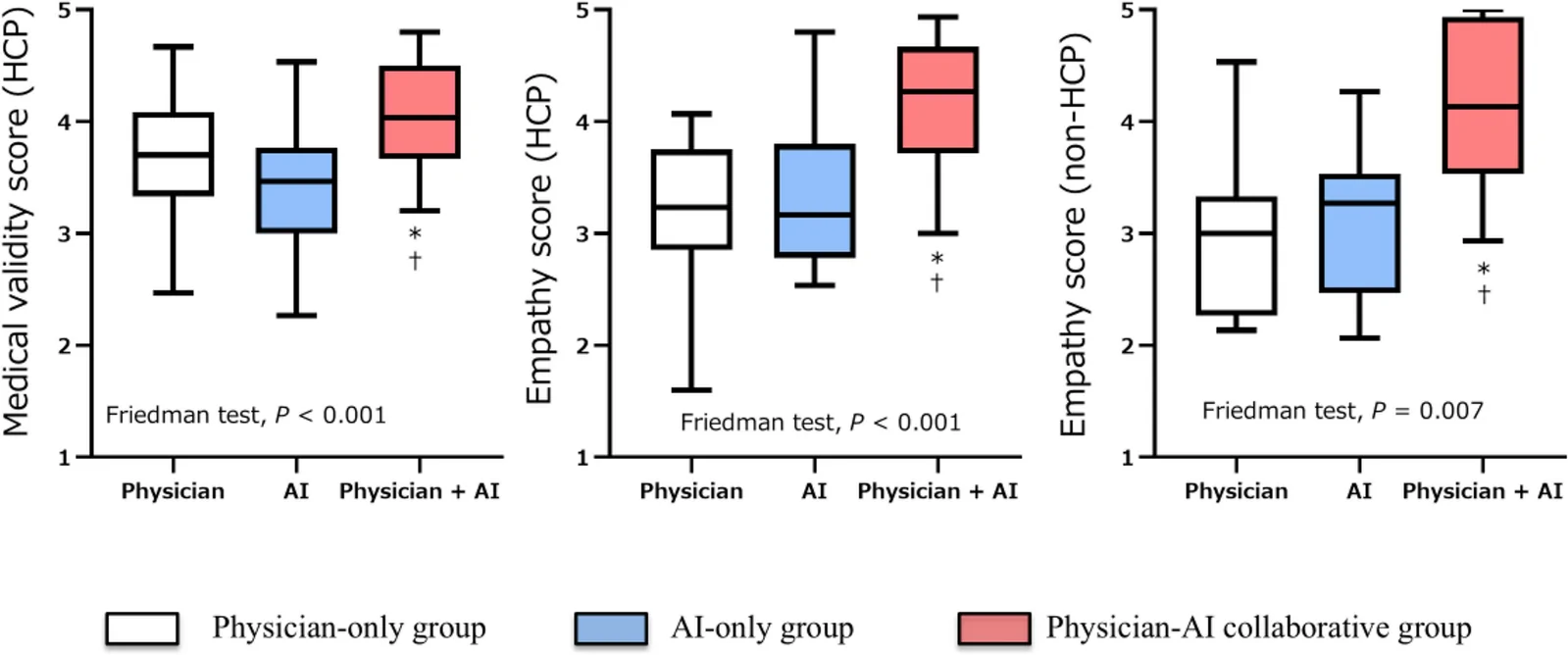
Prespecified primary outcome scores across the three response groups. Box plots showing medical validity rated by healthcare professionals, empathy rated by healthcare professionals, and empathy rated by nonhealthcare professionals across the physician-only, artificial intelligence (AI)-only, and physician–AI collaborative groups. The solid line within the box represents the median, the box represents the interquartile range, and the whiskers represent the lower and upper extremes. *P < 0.05 versus the physician-only group; †*P* < 0.05 versus the AI-only group

Participant characteristics are shown in Table 1, and participant-level mean scores are presented in Table 2 as medians. Friedman’s test showed significant between-group differences across all prespecified primary outcomes, including medical validity (*P* < 0.001) and empathy *(P* < 0.001) rated by healthcare professionals, and empathy (*P* = 0.007) rated by nonhealthcare professionals (Fig. 2). The corresponding Kendall’s W values were 0.64 (95% CI 0.49–0.80) for medical validity rated by healthcare professionals, 0.70 (95% CI 0.54–0.80) for empathy rated by healthcare professionals, and 0.33 (95% CI 0.08–0.76) for empathy rated by nonhealthcare professionals. Inter-rater agreement was limited across the three co-primary outcomes. The single-measure ICCs were 0.116 (95% CI 0.073–0.186) for medical validity rated by healthcare professionals, 0.283 (95% CI 0.203–0.397) for empathy rated by healthcare professionals, and 0.282 (95% CI 0.195–0.403) for empathy rated by nonhealthcare professionals (Additional File 4: Table S1).

**Table 1:** Participant characteristics

Characteristics	Healthcare professionals (*n* = 30)		Nonhealthcare professionals (*n* = 15)
	Physicians (*n* = 15)	Nurses (*n* = 15)	
Age, y	34.0 (32.0–37.5)	34.5 (31.3–45.0)	48.0 (37.5–55.5)
Male sex, *n* (%)	12 (80.0)	3 (20.0)	7 (46.7)
Department, *n* (%)	Surgery, 5 (33.3)	Operating room, 5 (33.3)	–
	Anesthesiology, 4 (26.7)	General ward, 4 (26.7)	
	Internal medicine, 3 (20.0)	Other, 3 (20.0)	
	Other, 2 (13.3)	Emergency department, 2 (13.3)	
	Emergency medicine, 1 (6.7)	ICU, 1 (6.7)	
Clinical experience, y	8.0 (8.0–9.0)	13.5 (9.8–24.5)	–

*ICU* intensive care unit, – not applicable

Data are presented as median (interquartile range) for continuous variables and *n* (%) for categorical variables

**Table 2:** The prespecified primary outcomes by response group

Outcome	Physician-only	AI-only	Physician–AI collaborative	*P* value^a^	Kendall’s W (95% CI)^b^
Medical validity score (HCP)	3.7 (3.4–4.0)	3.5 (3.0–3.7)	4.0 (3.7–4.4)	**<0.001**	0.64 (0.49–0.80)
Empathy score (HCP)	3.2 (2.8–3.7)	3.2 (2.8–3.8)	4.3 (3.7–4.7)	**<0.001**	0.70 (0.54–0.80)
Empathy score (non-HCP)	3.0 (2.4–3.0)	3.3 (2.7–3.5)	4.1 (3.6–4.7)	**0.007**	0.33 (0.08–0.76)

Bold values indicate statistical significance

Data are presented as median (interquartile range)

*HCP* healthcare professionals, *non-HCP* nonhealthcare professionals, *CI* confidence interval

^a^*P* values were obtained using the Friedman test and interpreted using a Bonferroni-adjusted significance threshold of 0.0167 for the three co-primary outcomes. Medical validity was assessed by healthcare professionals only.

^b^Kendall’s W was calculated as an effect size for the Friedman test, and 95% confidence intervals were estimated using 10,000 participant-level bootstrap samples

For medical validity rated by healthcare professionals (*n* = 30), the primary analysis showed that physician–AI collaborative responses received significantly higher ratings than both physician-only and AI-only responses (physician–AI collaboration vs. physician-only, *P* < 0.001; physician–AI collaboration vs. AI-only, *P* < 0.001), whereas no significant difference was observed between physician-only and AI-only responses (*P* = 0.210) (Table 3). The sensitivity analysis supported the higher ratings of physician–AI collaborative responses compared with physician-only responses (estimate, 0.42; 95% CI 0.33–0.50; *P* < 0.001). In contrast to the primary analysis, AI-only responses received lower ratings than physician-only responses in this model (estimate, − 0.17; 95% CI − 0.26 to − 0.08; *P* < 0.001) (Table 4). In the exploratory analysis, the estimated proportions of scores ≥ 4 were 85.4%, 64.6%, and 53.3% in the physician–AI collaborative, physician-only, and AI-only groups, respectively (Table 5). The physician–AI collaborative group had higher odds of receiving a score ≥ 4 than both other groups, and the physician-only group had higher odds than the AI-only group (Table 6). Thus, the higher ratings of physician–AI collaborative responses were consistent across analyses, whereas the difference between physician-only and AI-only responses depended on the analytic approach.

**Table 3:** Post hoc pairwise comparisons for the prespecified primary outcomes using Dunn’s multiple comparison test

Outcome	Comparison	*P* value^a^
Medical validity score (HCP)	Physician–AI collaborative vs. physician-only	**<0.001**
	Physician–AI collaborative vs. AI-only	**<0.001**
	Physician-only vs. AI-only	0.210
Empathy score (HCP)	Physician–AI collaborative vs. physician-only	**<0.001**
	Physician–AI collaborative vs. AI-only	**<0.001**
	Physician-only vs. AI-only	>0.999
Empathy score (non-HCP)	Physician–AI collaborative vs. physician-only	**0.019**
	Physician–AI collaborative vs. AI-only	**0.019**
	Physician-only vs. AI-only	>0.999

Bold values indicate statistical significance

Data are presented as *P* values from Dunn’s multiple comparison test

*HCP* healthcare professionals, *non-HCP* nonhealthcare professionals, *AI* artificial intelligence

^a^Dunn’s multiple comparison test was performed as a post hoc analysis following a significant Friedman test for each prespecified primary outcome. Statistical significance was defined as a two-sided *P* < 0.05. Medical validity was assessed by healthcare professionals only

**Table 4:** Sensitivity analysis using linear mixed-effects models for the prespecified primary outcomes

Outcome	Comparison	Estimate (95% CI)^a^	*P* value^b^
Medical validity score (HCP)	Physician–AI collaborative vs. physician-only	0.42 (0.33–0.50)	**<0.001**
	AI-only vs. physician-only	−0.17 (−0.26 to −0.08)	**<0.001**
Empathy score (HCP)	Physician–AI collaborative vs. physician-only	0.98 (0.86–1.10)	**<0.001**
	AI-only vs. physician-only	0.12 (0.00–0.24)	0.054
Empathy score (non-HCP)	Physician–AI collaborative vs. physician-only	1.21 (1.03–1.40)	**<0.001**
	AI-only vs. physician-only	0.22 (0.04–0.40)	**0.016**

Bold values indicate statistical significance

*CI* confidence interval, *HCP* healthcare professionals, *non-HCP* nonhealthcare professionals, *AI* artificial intelligence

^a^Estimates and 95% confidence intervals from linear mixed-effects models. The physician-only group was used as the reference category. Positive estimates indicate higher scores in the first-named group

^b^*P* values from linear mixed-effects models

**Table 5:** Model-estimated proportions of high ratings for medical validity and empathy across response groups

Outcome	Physician-only, % (95% CI)^a^	AI-only, % (95% CI)^a^	Physician–AI collaborative, % (95% CI)^a^
Medical validity score (HCP)	64.6 (48.0–78.3)	53.3 (36.6–69.2)	85.4 (74.4–92.1)
Empathy score (HCP)	43.0 (30.0–57.1)	42.1 (29.3–56.2)	85.2 (76.1–91.2)
Empathy score (non-HCP)	30.3 (21.5–40.9)	35.6 (25.9–46.6)	75.9 (66.1–83.5)

*CI* confidence interval, *HCP* healthcare professionals, *non-HCP* nonhealthcare professionals, *AI* artificial intelligence

^a^Proportions and 95% confidence intervals were estimated using generalized linear mixed-effects models. Scores ≥ 4 were defined as high ratings

**Table 6:** Odds ratios for high ratings of medical validity and empathy across response groups

Outcome	Comparison	OR (95% CI)^a^	*P* value
Medical validity score (HCP)	AI-only vs. physician-only	0.63 (0.42–0.93)	**0.005**
	Physician–AI collaborative vs. physician-only	3.20 (2.07–4.90)	**<0.001**
	Physician–AI collaborative vs. AI-only	5.10 (3.30–7.94)	**<0.001**
Empathy score (HCP)	AI-only vs. physician-only	0.96 (0.67–1.39)	0.810
	Physician–AI collaborative vs. physician-only	7.63 (4.93–11.79)	**<0.001**
	Physician–AI collaborative vs. AI-only	7.94 (5.10–12.22)	**<0.001**
Empathy score (non-HCP)	AI-only vs. physician-only	1.27 (0.77–2.08)	0.250
	Physician–AI collaborative vs. physician-only	7.25 (4.22–12.36)	**<0.001**
	Physician–AI collaborative vs. AI-only	5.68 (3.36–9.62)	**<0.001**

Bold values indicate statistical significance

*OR* odds ratio, *CI* confidence interval, *HCP* healthcare professionals, *non-HCP* nonhealthcare professionals, *AI* artificial intelligence

^a^Odds ratios and 95% confidence intervals were estimated using generalized linear mixed-effects models. Scores ≥ 4 were defined as high ratings. An odds ratio > 1 indicates higher odds in the first-named group, whereas an odds ratio < 1 indicates higher odds in the second-named group

For empathy rated by healthcare professionals (*n* = 30), the primary analysis showed that physician–AI collaborative responses received significantly higher ratings than both physician-only and AI-only responses (physician–AI collaboration vs. physician-only, *P* < 0.001; physician–AI collaboration vs. AI-only, *P* < 0.001), whereas physician-only and AI-only responses did not differ significantly (*P* > 0.999) (Table 3). The sensitivity analysis supported the higher ratings of physician–AI collaborative responses compared with physician-only responses (estimate, 0.98; 95% CI 0.86–1.10; *P* < 0.001). No significant difference was observed between AI-only and physician-only responses (estimate, 0.12; 95% CI 0.00–0.24; *P* = 0.054) (Table 4). In the exploratory analysis, the estimated proportions of scores ≥ 4 were 85.2%, 43.0%, and 42.1% in the physician–AI collaborative, physician-only, and AI-only groups, respectively (Table 5). The physician–AI collaborative group had higher odds of receiving a score ≥ 4 than both other groups, whereas the physician-only and AI-only groups did not differ significantly (Table 6). These analyses consistently supported higher empathy ratings for physician–AI collaborative responses, with no clear difference between physician-only and AI-only responses.

For empathy rated by nonhealthcare professionals (*n* = 15), the primary analysis showed that physician–AI collaborative responses received significantly higher ratings than both physician-only and AI-only responses (physician–AI collaboration vs. physician-only, *P* = 0.019; physician–AI collaboration vs. AI-only, *P* = 0.019), whereas physician-only and AI-only responses did not differ significantly (*P* > 0.999) (Table 3). The sensitivity analysis supported the higher ratings of physician–AI collaborative responses compared with physician-only responses (estimate, 1.21; 95% CI 1.03–1.40; *P* < 0.001). In contrast to the primary analysis, AI-only responses received slightly higher ratings than physician-only responses in this model (estimate, 0.22; 95% CI 0.04–0.40; *P* = 0.016) (Table 4). In the exploratory analysis, the estimated proportions of scores ≥ 4 were 75.9%, 30.3%, and 35.6% in the physician–AI collaborative, physician-only, and AI-only groups, respectively (Table 5). The physician–AI collaborative group had higher odds of receiving a score ≥ 4 than both other groups, whereas physician-only and AI-only responses did not differ significantly (Table 6). Thus, the higher ratings of physician–AI collaborative responses were consistent across analyses, whereas the small difference between AI-only and physician-only responses was observed only in the sensitivity analysis.

Across the three prespecified primary outcomes, the primary analyses showed that physician–AI collaborative responses received higher ratings than both physician-only and AI-only responses, a pattern also observed in the sensitivity and exploratory analyses. By contrast, comparisons between physician-only and AI-only responses varied across analytic approaches: no significant differences were observed for any outcome in the primary analyses, whereas a difference in medical validity was observed in both the sensitivity and exploratory analyses, and a difference in empathy rated by nonhealthcare professionals was observed only in the sensitivity analyses.

In the post hoc response-length analysis, median character counts were 336 (IQR, 259–531), 234 (IQR, 181–248), and 499 (IQR, 283–645) for physician-only, AI-only, and physician–AI collaborative responses, respectively. Both physician-only and physician–AI collaborative responses were longer than AI-only responses, whereas the former two did not differ (Additional File 5: Tables S2 and S3). After adjustment for response length, the estimated between-group differences were materially unchanged from the unadjusted sensitivity analyses, and character count was not associated with any primary outcome (Additional File 6: Table S4).

As a secondary analysis, the group-specific slopes (*β*) were 0.29 in the physician-only group (95% CI 0.24–0.34; *P* < 0.001), 0.38 in the AI-only group (95% CI 0.32–0.44; *P* < 0.001), and 0.41 in the physician–AI collaborative group (95% CI 0.34–0.48; *P* < 0.001). These findings indicate a positive association between empathy and medical validity across all groups (Table 7).

**Table 7:** Association between medical validity and empathy among healthcare professionals

Response group	*β* (95% CI)^a^	*P* value
physician-only	0.29 (0.24–0.34)	**<0.001**
AI-only	0.38 (0.32–0.44)	**<0.001**
Physician–AI collaborative	0.41 (0.34–0.48)	**<0.001**

Bold values indicate statistical significance

*β* regression coefficient, *CI* confidence interval, *AI* artificial intelligence

^a^*β* coefficients and 95% confidence intervals were obtained from linear mixed-effects models. Higher *β* values indicate a stronger positive association between medical validity and empathy within each response group

## Discussion

This study compared written explanations generated by physicians alone, an LLM-based chatbot alone, and physicians who revised LLM-generated drafts in simulated ICU EOL scenarios. In the primary analyses, physician–AI collaborative responses received higher ratings than both physician-only and AI-only responses across all three primary outcomes. However, because only the final responses were evaluated, the contributions of the LLM-generated draft, physician revision, and the two-stage drafting-and-revision process could not be distinguished. The findings should therefore be interpreted as an association between the collaborative workflow as a whole and higher ratings, rather than evidence of a specific contribution of the LLM-generated draft.

In the primary analyses, the difference between the participant-level median scores of the physician–AI collaborative and physician-only groups was 0.3 points for medical validity and 1.1 points for empathy rated by both healthcare and nonhealthcare professionals. Thus, the difference was more pronounced for perceived empathy than for medical validity. However, because no validated threshold for a clinically meaningful difference was available for these investigator-developed 5-point scales, the clinical importance of these differences remains uncertain.

Physician–AI collaborative responses received the highest ratings for medical validity. However, because no board-certified intensivists were included among the healthcare professional raters, these findings should be interpreted as assessments by clinicians from diverse specialties and clinical settings rather than definitive specialist assessments of ICU EOL communication. Previous research has suggested that LLM-generated informed consent documentation may provide more comprehensive coverage of essential information [20]. However, we did not systematically compare the initial LLM-generated drafts with the final physician-revised responses or characterize the nature and extent of the physicians’ revisions. Therefore, correction of inaccurate information, addition of clinically relevant details, incorporation of context-specific information, and improved organization remain possible explanations rather than demonstrated mechanisms.

For empathy, physician–AI collaborative responses received the highest ratings from both healthcare and nonhealthcare professionals. However, among the 15 nonhealthcare professionals, Kendall’s W was smaller and its 95% CI wider than among healthcare professionals; this finding should therefore be interpreted cautiously. Previous studies have reported high perceived empathy in LLM-generated responses [10, 11, 21]. Physician revision of LLM-generated drafts may have improved empathy by incorporating the patient’s background and using more supportive language. However, because the revisions were not analyzed, the reasons for the higher empathy ratings remain unclear.

Physician-only and physician–AI collaborative responses were longer than AI-only responses, whereas the former two did not differ. Adjusting for character count did not materially alter the between-group estimates, and character count was not associated with any primary outcomes. Thus, response length alone did not explain the higher ratings of collaborative responses, although other textual features may have contributed.

Differences between physician-only and AI-only responses were inconsistent across analytic approaches. In the sensitivity analyses, AI-only responses received slightly lower medical validity ratings from healthcare professionals but slightly higher empathy ratings from nonhealthcare professionals than physician-only responses. These discrepancies may reflect differences in analytic approaches and data structures; however, because the estimated differences were small and no validated thresholds for clinically meaningful differences were available, they should be interpreted cautiously.

### Limitations

This study has several limitations. First, the scenario-based design limited the evaluation of real-world ICU communication because written scenarios did not capture nonverbal communication or the emotional context of actual family conferences. Second, empathy ratings from only 15 nonhealthcare professionals yielded a smaller Kendall’s W and wider uncertainty than those from healthcare professionals and should therefore be interpreted with greater caution. These ratings provide an indirect reflection of the perspectives of patients and families rather than direct evidence of benefit to actual patients or families. Third, this single-center study evaluated only three ICU scenarios using one LLM version, one predefined prompt, and one generated response per question. Because variability across repeated generations was not assessed, the reproducibility and range of possible LLM-generated outputs remain uncertain. Generalizability to other settings, alternative prompt wording, models, model versions, and outputs is also limited. Fourth, the evaluation procedure may have introduced several biases. The substantial rating burden may have caused fatigue, and fixed question order may have introduced order effects. Simultaneous presentation may have promoted comparative rather than independent judgments and amplified perceived between-group differences. Despite randomized response order and concealed group labels, stylistic features may have revealed the response-generation procedure, and neither blinding success nor attitudes toward AI were assessed. Fifth, because different physicians generated the physician-only and physician–AI collaborative responses, differences in writing ability may have influenced the results. Sixth, the study-specific medical validity scale was not formally validated, and the CARE Measure, which is not validated for text-only assessment, was used only as a conceptual guide. Inter-rater agreement was limited across all three primary outcomes. No board-certified intensivists were included among the raters; healthcare professional ratings therefore reflect assessments by heterogeneous clinicians rather than specialist assessments of ICU EOL communication. Seventh, the content and extent of physician revisions and the time and effort required were not assessed. Therefore, the factors contributing to the higher ratings and the efficiency of the collaborative workflow remain unclear. Eighth, no process-matched control was included in which one physician’s draft was reviewed and revised by another. Thus, the contribution of the LLM-generated draft could not be distinguished from the general effect of a two-stage drafting-and-revision process.

## Conclusions

In this scenario-based study of communication in ICU EOL care, physician–AI collaborative responses received the highest ratings, with more pronounced differences for empathy than for medical validity. However, the specific contribution of the LLM-generated draft could not be distinguished from subsequent physician revision or the general effect of a two-stage drafting-and-revision process. Further multicenter, process-matched prospective studies are warranted.

## Supplementary Information

Additional file 1. Clinical scenarios used in this study. This file contains the full text of the three clinical scenarios simulating end-of-lifecare in the intensive care unit. The scenarios cover septic shock requiring veno-arterial extracorporeal membrane oxygenation, severe respiratory failure in a patient with malignancy, and postcardiac arrest syndrome

Additional file 2. Full set of physician-only, artificial intelligence-only, and physician–artificial intelligence collaborative responses. This file contains the complete response texts for all 15 questions across the three clinical scenarios. For each question, responses from the physician-only, artificial intelligence-only, and physician–AI collaborative groups are provided

Additional file 3. Prompt entered into ChatGPT for response generation. This file contains the predefined prompt entered into ChatGPT for each question in each clinical scenario. This prompt was used to generate the artificial intelligence-only responses and the initial drafts for the physician–AI collaborative group

Additional file 4. Inter-rater reliability for the three prespecified primary outcomes. This file contains the single-measure intraclass correlation coefficients and 95% confidence intervals for the three prespecified primary outcomes

Additional file 5. Comparison of response length across the three response groups. This file contains descriptive statistics for response length across the physician-only, AI-only, and physician–AI collaborative groups, as well as the results of the overall and post hoc pairwise comparisons of response length

Additional file 6. Response-length-adjusted sensitivity analyses. This file contains the results of additional post hoc analyses using linear mixed-effects models accounting for response length. For each co-primary outcome, Table S4 presents the estimated effects of response group and mean-centered character count, expressed per 100 Japanese characters, with 95% confidence intervals and *P* values.

## Data Availability

The datasets used and/or analyzed during the current study are available from the corresponding author on reasonable request.

## Abbreviations

EOL: End-of-life
ICU: Intensive care unit
AI: Artificial intelligence
LLM: Large language model
CARE: Consultation and relational empathy
CI: Confidence interval
ICC: Intraclass correlation coefficient
OR: Odds ratio
