# Supplementary material for: Physician-revised AI-generated drafts are associated with higher ratings of written explanations in end-of-life care in the intensive care unit: a scenario-based single-center cross-sectional study
Source: J Intensive Care. 2026 Aug 24;14:66. doi: 10.1186/s40560-026-00930-2 (PMC13501828; doi:10.1186/s40560-026-00930-2)
Supplement: Supplementary file 1 [file 40560_2026_930_MOESM1_ESM.docx]

Additional File 1. Clinical scenarios used in this study

[Scenario1: septic shock requiring veno-arterial extracorporeal membrane oxygenation]

The following describes the patient’s premorbid information.

The patient was in their 60s and had no history of major illness or currently diagnosed medical conditions. The patient was independent in all activities of daily living. The patient lived with their spouse and had two children. The patient worked as a barber and enjoyed park golf.

The patient initially visited a nearby hospital because of sudden onset of high fever, chills, and diarrhea. They were diagnosed with gastroenteritis, prescribed antipyretic medication, and sent home for observation. However, the symptoms did not improve by the following day, and the patient gradually became drowsy and less responsive, prompting an emergency ambulance call. On arrival at the emergency department of Hospital A, the patient’s level of consciousness had further deteriorated, and hypotension, tachycardia, and tachypnea were noted. Based on the examination findings, fulminant streptococcal toxic shock syndrome was considered the most likely diagnosis, along with associated sepsis. Regarding respiration, the patient’s oxygenation was temporarily maintained with the highest possible oxygen flow delivered via an oxygen mask. However, with respect to circulation, it remained difficult to maintain blood pressure despite intravenous fluid resuscitation and the use of vasopressor agents. To ensure adequate systemic oxygen delivery, endotracheal intubation was performed and mechanical ventilation was initiated. In addition, because the patient’s blood pressure could not be maintained, the medical team determined that treatment with veno-arterial extracorporeal membrane oxygenation (V-A ECMO) was necessary as a life-sustaining measure. V-A ECMO is a life-support system that temporarily provides circulatory and respiratory support by functioning as an artificial heart and lung. Accordingly, the patient was transferred to Hospital B, where advanced intensive care could be provided.

Because of sedation, the patient was unable to respond when spoken to. The patient’s spouse and children arrived at the intensive care unit (ICU) of Hospital B. At that time, the patient was in an extremely critical condition, requiring urgent initiation of V-A ECMO.

Q1. The patient’s family appears highly distressed and says to you (the intensivist), “Is my spouse going to be okay? Please do whatever you can to save them.” Please provide a clear and empathetic explanation of the patient’s current condition, the necessity of V-A ECMO, its potential complications, and the predicted clinical course.

After understanding the explanation, the patient’s family consented to treatment with V-A ECMO. Cannulas were then inserted into the femoral artery and vein, and V-A ECMO support was initiated. The initiation of ECMO was completed smoothly, without any procedural complications. After ECMO was started, the patient’s blood pressure and respiratory status stabilized. Antimicrobial therapy was administered to treat the underlying bacterial infection causing sepsis, and circulatory and respiratory support with V-A ECMO and mechanical ventilation was continued. Transthoracic echocardiography showed severely impaired cardiac function, likely attributable to the effects of sepsis.

Despite 10 days of continued antimicrobial therapy, the patient’s cardiac function did not improve, and the patient remained dependent on life-sustaining support, including ECMO and mechanical ventilation, which could not be discontinued. After repeated multidisciplinary discussions involving intensivists, nurses, clinical engineers, pharmacists, and physical therapists, the team concluded that multiple organs had sustained severe damage and that the likelihood of recovery was exceedingly low, even with continued treatment. The patient was deeply sedated and unable to communicate. You repeatedly explained to the family that the patient’s condition was extremely critical and that the likelihood of recovery was exceedingly low, and the family demonstrated a clear understanding of the situation. During these discussions, the following information was obtained from the family: the patient had no history of serious illness and had never discussed preferences regarding life-sustaining treatment in the event of a life-threatening condition. The family also described the patient as someone who greatly valued playing park golf on weekends and took pride in working as a barber, particularly enjoying conversations with regular customers.

Q2. If the likelihood of clinically meaningful recovery becomes extremely low, you may need to consider discontinuing ECMO support. You also need to discuss in advance whether to attempt cardiopulmonary resuscitation, including chest compressions and defibrillation, if the patient’s heart stops. In some situations, life-sustaining therapies such as ECMO and resuscitation efforts may not benefit the patient and may instead increase suffering. Please communicate these considerations with compassion, answer any questions, and help clarify the patient’s and family’s values and preferences to guide the most appropriate plan of care.

The family understood your explanation. The patient’s spouse then said, “I do not think my spouse would have wanted to remain alive connected to tubes if there were no chance of recovery.” However, the spouse’s expression suggested uncertainty, and you gently asked, “Could you please tell me how you honestly feel at this moment?”

Q3. The patient’s spouse remarked, “I understand what you are saying, but part of me still hopes for a miracle,” indicating that they had not yet fully relinquished hope. Please speak to the patient’s family clearly and empathetically, taking their feelings into account.

After repeated discussions between the medical team and the family, it was decided that ECMO would be withdrawn and would not be reinitiated even if the patient developed worsening respiratory failure, hypotension, or cardiac arrest. It was also agreed that cardiopulmonary resuscitation, including chest compressions, would not be performed in the event of cardiac arrest. The plan was therefore shifted toward comfort-focused care, with the goal of relieving suffering as much as possible and allowing the patient to spend meaningful time with family until the end of life.

Thereafter, the patient’s spouse began spending increasing amounts of time at the bedside, including late at night. Except during nursing procedures, the spouse remained continuously by the patient’s side and appeared profoundly fatigued. According to the nursing staff, because the spouse lived far from the hospital, they had been spending the past several days sleeping in a car near the hospital. When you visited the patient at the bedside, the spouse said, “Thank you, as always, for everything you have done.”

Q4. The patient’s spouse appeared profoundly fatigued after a prolonged period of bedside attendance and was occasionally tearful while holding the patient’s hand and watching over the patient. The spouse remarked, “Today, my spouse appears to have a slightly better facial expression.” Considering the psychological burden on the patient’s spouse, you spoke in a clear and empathetic manner.

The patient’s condition worsened further the following day, and the patient passed away peacefully in the presence of family. The family expressed their gratitude to the medical team.

Q5. The patient’s spouse stated, “I am truly grateful to all of you. However, I still occasionally question whether the decisions we made were truly the right ones.” You then responded in a clear and empathetic manner.

[Scenario2: severe respiratory failure in a patient with malignancy]

The following describes the patient’s premorbid information.

The patient was in their 50s, had no history of serious illness, and had been independent in activities of daily living. The patient lived with their spouse and had one child. The patient worked as a police officer and enjoyed traveling as a hobby.

More recently, the patient developed symptoms including fatigue, palpitations, and shortness of breath, and visited a nearby hospital. Blood tests revealed anemia and a markedly elevated white blood cell count, raising suspicion of a hematologic disorder, and the patient was therefore referred to Hospital C. Further evaluation revealed that the patient had acute myeloid leukemia, a hematologic malignancy, and the patient was admitted to the hospital to begin chemotherapy. On the third day of treatment, the patient developed dyspnea, and chest radiography showed abnormal bilateral pulmonary opacities. Leukemic infiltration of the lungs was considered the most likely cause of the worsening respiratory status. Despite administration of high-flow oxygen via face mask, the patient remained hypoxemic and appeared markedly tachypneic and distressed. Because the patient was in critical respiratory failure, transfer to the ICU was required.

After admission to the ICU, the patient’s respiratory status deteriorated further, and the sensation of dyspnea became more severe. We therefore proposed endotracheal intubation and initiation of mechanical ventilation to support breathing, and the patient provided informed consent. Although the family was on the way to the hospital, there was not enough time to wait for their arrival. Therefore, the patient’s condition and the proposed treatment were explained to the family by telephone, and they also consented to endotracheal intubation.

By that time, the patient had already undergone endotracheal intubation and was unable to respond because of the effects of the sedative medications used during the procedure. Shortly thereafter, the patient’s family arrived at the ICU.

Q1. The patient’s spouse, appearing extremely distressed, said to you (the intensivist), “Why has my spouse’s condition suddenly worsened to this extent? Please do anything possible to save them.” In response, you explained the patient’s current condition, the reasons for endotracheal intubation and mechanical ventilation, and the expected clinical course in a clear and empathetic manner.

Despite continued respiratory support with mechanical ventilation, the patient’s respiratory status gradually worsened. Although chemotherapy would ordinarily be required to eradicate the leukemic cells in the blood and lungs, the patient’s general condition was too poor to allow initiation of such treatment. Despite ongoing ventilatory support, the patient continued to show signs of severe respiratory distressed despite sedation. After repeated multidisciplinary discussions, the team concluded that, because definitive treatment with chemotherapy could not be provided, the likelihood of liberation from mechanical ventilation was exceedingly low and that further continuation of treatment would only prolong the patient’s suffering. During these discussions, the hematology team also explained that, if chemotherapy could not be initiated, the expected prognosis would be only a few days to about one week.

Although the patient was intubated, communication by writing remained possible with adjustment of the sedative dose. During the course of treatment, the following information was obtained from the patient and family: the family stated that the patient had never experienced any serious illness and that they had never discussed what should be done in the event of a life-threatening condition. The patient worked as a police officer, had a strong sense of justice, and took great pride in the job. The patient enjoyed traveling as a hobby and took trips with their spouse several times a year. They had also planned to travel around the world with their spouse after retirement and regarded this as a major source of purpose and motivation in life.

Q2. You explained the patient’s condition since admission to the ICU and the expected clinical course in a clear and empathetic manner to the patient and family.

The patient and family understood your explanation. The patient then clearly expressed a wish not to undergo any further treatment, including chemotherapy, stating, “I am suffering, and I do not want any more treatment such as chemotherapy. I would like to be kept comfortable and free from distress at the end of life.” However, the family pleaded for continued treatment, saying, “Please do not say that. Try a little longer. You will surely get better.” A discrepancy had therefore emerged between the wishes of the patient and those of the family regarding further treatment, and the patient appeared distressed and conflicted.

Q3. You are now asked to explain the patient’s treatment plan once again to the patient and the family, from both the medical perspective and the patient’s own wishes. While being mindful of the family’s feelings, please provide a clear and empathetic explanation of the future treatment plan, with the greatest possible respect for the patient’s autonomy, including the transition to palliative-focused care.

However, the spouse said, “I understand your explanation. But my spouse is normally a very strong and determined person and is not someone who would easily say such things. I think they are confused right now and unable to make a sound decision. Please do whatever you can to save them.”

Meanwhile, the patient wrote to you, “I am grateful that my family is encouraging me to keep fighting, but if I say that I cannot go on any longer, would that mean I am betraying them?”

Q4. When responding to the patient’s questions, please offer supportive and empathetic words in a clear and understandable manner, while being attentive to the patient’s feelings of guilt and emotional conflict.

After repeated discussions among the medical team, the patient, and the family, it was decided to respect the patient’s wishes and transition to palliative care. In other words, no further curative treatment, including chemotherapy, would be provided, and the focus of care would shift to relieving the patient’s suffering and supporting the patient in living as meaningfully and comfortably as possible until the end of life.

Q5. At a later time, the family member said, “Thank you very much for everything you have done for us. At first, I was unable to accept the reality of the situation, and I found it difficult to accept their wishes to discontinue treatment. I even said to them, ‘Keep fighting’.” Please speak to the patient’s family in a way that is both clear and empathetic.

The patient passed away peacefully the following day in the presence of family.

[Scenario3: post-cardiac arrest syndrome]

The following describes the patient’s premorbid information.

The patient was in their 80s and had no history of major illness. The patient had been receiving regular care at a nearby hospital for hypertension, diabetes mellitus, and dyslipidemia. The patient was independent in activities of daily living. The patient lived with the spouse, and they had one child. The patient was retired and had previously worked as a high school teacher. The patient’s hobby was home gardening.

One day, while shopping at a supermarket near their home, the patient suddenly lost consciousness and collapsed. A nearby bystander called out to the patient but received no response, so an ambulance was immediately called and cardiopulmonary resuscitation was initiated. Approximately ten minutes later, an ambulance arrived. After the emergency medical team attached an automated external defibrillator and delivered one shock, spontaneous circulation was restored. However, the patient had still not regained consciousness. The patient was then transported by ambulance to the ICU of Hospital D.

In the ICU, the patient’s blood pressure and respiratory status were stable; however, consciousness had not yet returned, and the patient did not respond to verbal commands or physical stimulation. Because the patient remained completely unconscious and was at high risk of aspiration, an endotracheal tube was inserted and mechanical ventilation was initiated. Further evaluation revealed no obvious abnormalities, including in the heart. However, the patient remained unconscious, and computed tomography (CT) of the head showed cerebral edema, likely due to hypoxic brain injury following the cardiac arrest. At that time, the patient’s family arrived at the ICU.

Q1. The patient’s son or daughter, with a look of mixed anxiety and hope, asked you (the intensivist), “Since the heart has started beating again, does that mean they will be all right? Will they recover and become well again?” Please provide a clear and empathetic explanation of the patient’s current condition, the treatment options to be considered from this point forward, including targeted temperature management, and the expected clinical course.

To reduce the risk of further brain injury as much as possible, the family was informed about targeted temperature management, a treatment that lowers the patient’s body temperature, and consent was obtained. The plan was to prevent any increase in body temperature for at least 72 hours after the return of spontaneous circulation, after which neurological function would be reassessed. In addition, the cause of the cardiac arrest remained unclear, and no cause-specific treatment could be provided at that time.

Three days later, targeted temperature management was discontinued; however, the patient showed no recovery of consciousness. Subsequent head CT and electroencephalography revealed severe brain injury. After repeated multidisciplinary discussions, the team concluded that the likelihood of recovery of consciousness was extremely low. During this clinical course, the family also provided the following information: the patient and the spouse had discussed, several years earlier, what they would want if either of them became seriously ill. They had agreed that, if recovery was unlikely after a major illness or if either of them lost consciousness, they would forgo life-prolonging treatment and allow the illness to follow its natural course. The patient had often said they had lived a full life, that their child had grown up and was living well independently, and that they had no major regrets. As both of them had become less steady on their feet, they no longer went out often, and in recent years they had been enjoying spending time together working in their home garden.

Q2. You are asked to explain to the patient’s family, in a clear and empathetic manner, the patient’s condition after targeted temperature management, the examination findings, and the expected clinical course.

The patient’s family appeared to have understood the explanation well. The patient’s spouse said, “Some time ago, we talked about how we had both lived full lives, and that if either of us became seriously ill, we would let nature take its course. The patient had done more than enough, and I do not think that the patient should continue to undergo burdensome treatment any longer.” It also appeared that the patient had wished to spend their final moments peacefully, surrounded by family. However, the patient’s son or daughter expressed a desire to continue treatment, saying, “But the possibility of recovery is not zero, is it? Please do everything that can be done.” As a result, there was a discrepancy within the family regarding the goals of care.

Q3. The spouse and children have differing views and have not yet been able to decide on the treatment plan. You are asked to explain the future course of treatment to the family in a clear and empathetic manner, while respecting the family’s feelings and addressing both the patient’s wishes and the medical perspective.

After repeated discussions between the medical team and the patient’s family, it was decided to respect the patient’s previously expressed wish and to focus on palliative care, without pursuing any further treatment. The patient’s blood pressure, oxygenation, and other vital parameters, apart from consciousness, remained stable, and it was considered possible that the patient might survive for several more months. At present, the patient is intubated with an endotracheal tube inserted through the mouth. However, prolonged transoral intubation is associated with an increased risk of complications. Therefore, tracheostomy, in which a breathing tube is inserted directly through the neck into the trachea, was considered necessary.

Q4. You are now going to explain to the patient’s family why a tracheostomy is necessary. Please explain, in a clear and empathetic manner, the reasons for performing a tracheostomy in the current situation, as well as its advantages and disadvantages.

The patient’s family fully understood the need for tracheostomy and provided consent. The procedure was successfully performed the following day. As the patient’s respiratory status remained stable, ventilatory support was no longer required, and only the tracheostomy tube remained in place.

On a later visit, when the patient’s spouse came to see the patient, the patient’s spouse became tearful and expressed anxiety about the future, saying, “What will happen from here? If the patient never regains consciousness, is there still any meaning in our being by their side and speaking to them?”

Q5. The patient’s family is deeply distressed by the possibility that the patient may not regain consciousness and feels uncertain about how to face and support the patient going forward. Please explain, in a clear and empathetic manner, how the family may continue to support the patient and remain by their side while acknowledging their grief and anxiety.
