# Supplementary material for: Physician-revised AI-generated drafts are associated with higher ratings of written explanations in end-of-life care in the intensive care unit: a scenario-based single-center cross-sectional study
Source: J Intensive Care. 2026 Aug 24;14:66. doi: 10.1186/s40560-026-00930-2 (PMC13501828; doi:10.1186/s40560-026-00930-2)
Supplement: Supplementary file 2 [file 40560_2026_930_MOESM2_ESM.docx]

Additional File 2. Full set of physician-only, AI-only, and physician-AI collaborative responses

[Scenario1: septic shock requiring veno-arterial extracorporeal membrane oxygenation]

Q1. The patient’s family appears highly distressed and says to you (the intensivist), “Is my spouse going to be okay? Please do whatever you can to save them.” Please provide a clear and empathetic explanation of the patient’s current condition, the necessity of veno-arterial extracorporeal membrane oxygenation (V-A ECMO), its potential complications, and the predicted clinical course.

Response 1 (Physician-only group)

The patient was emergently transported to Hospital A following an abrupt onset of illness and was subsequently diagnosed with a severe bacterial infection due to fulminant streptococcal toxic shock syndrome. In response to this bacterial infection, an exaggerated host immune response was elicited, resulting in systemic inflammation and subsequent progression to multi-organ dysfunction.

To treat this severe bacterial infection, antibiotic therapy has been initiated. However, antibiotic therapy alone is insufficient; concomitant supportive care is required to maintain systemic organ function, including respiratory support to ensure adequate oxygenation and circulatory support to preserve oxygen delivery throughout the body. With respect to respiratory management, the airway was secured by endotracheal intubation, and mechanical ventilation was initiated to provide ventilatory support. However, the patient has developed sepsis-associated cardiac dysfunction, and despite ventilatory support and adequate oxygenation, reduced cardiac output is limiting systemic oxygen delivery to vital organs, placing the patient in a life-threatening condition.

It is necessary to maintain adequate systemic oxygen delivery, similar to that under normal physiologic conditions. Therefore, extracorporeal cardiopulmonary support is considered necessary. ECMO provides extracorporeal gas exchange by draining deoxygenated, hypercapnic blood, enabling oxygenation and carbon dioxide removal, and returning oxygenated blood to the systemic circulation, thereby temporarily substituting for native cardiopulmonary function. You may have heard of ECMO during the COVID-19 pandemic. The patient remains in critical condition with severe cardiopulmonary failure. Despite mechanical ventilation, oxygenation and carbon dioxide clearance are inadequate. Furthermore, even with inotropic support, cardiac function remains profoundly impaired, resulting in inadequate systemic perfusion. Therefore, initiation of ECMO is deemed necessary to stabilize gas exchange and systemic perfusion and to support ongoing management. ECMO is associated with vascular access-related complications. Large-bore arterial and venous cannulas are inserted into the femoral artery and vein. Cannulation may cause vascular injury and bleeding. Moreover, distal perfusion of the cannulated limb may be compromised, with a risk of lower-extremity ischemia. Despite the potential complications associated with ECMO, the immediate priority in the current clinical context is to optimize cardiac function and stabilize hemodynamics.

Regarding subsequent management, ECMO will be used to provide temporary cardiopulmonary support and to reduce cardiopulmonary workload. During this period, antibiotic therapy will be continued to eradicate the causative organism. In parallel, comprehensive supportive care will be provided to mitigate the infection-associated hyperinflammatory response and facilitate resolution of systemic organ dysfunction. Once the infection-associated hyperinflammatory response subsides, recovery of cardiopulmonary function is anticipated. If cardiopulmonary function improves sufficiently, weaning from ECMO and mechanical ventilation can be pursued. The subsequent clinical course will largely depend on two key factors: whether antibiotic therapy can successfully eradicate the causative pathogen and whether the dysregulated host immune response can be adequately controlled.

Response 2 (AI-only group)

The patient has a severe systemic infection complicated by profound hypotension and subsequent cardiopulmonary dysfunction. Despite ongoing intravenous fluid resuscitation and vasoactive support, the patient remains hemodynamically unstable, and these measures alone have been insufficient to achieve hemodynamic stabilization.

Therefore, initiation of ECMO is required to provide cardiopulmonary support. ECMO provides cardiopulmonary support via femoral vascular cannulation, draining blood to an extracorporeal circuit for membrane oxygenation and returning oxygenated blood to the systemic circulation, thereby supporting both circulation and gas exchange. Potential complications include bleeding, thrombosis, and infection. However, in the absence of ECMO support, survival is unlikely.

If treatment is successful, the patient’s cardiac and pulmonary function may gradually recover. Nevertheless, given the severity of the underlying infection, prolonged intensive support may be required, and meaningful recovery may not be attainable. I recognize that this is an extremely distressing situation. We will make every effort to provide the best possible care, and we will continue to support you throughout this process.

Response 3 (Physician-AI collaborative group)

I understand that this sudden development has come as a great shock, and I fully appreciate your concern. Although it is extremely rare for an infection to become this severe, given the current situation, we will make every effort to provide the best possible treatment using all available measures. I will explain the patient’s current condition, the planned treatment, and the anticipated clinical course as clearly as possible.

The patient is currently in a critical condition with severe hypotension due to infection with fulminant group A streptococcus (commonly referred to as “flesh-eating bacteria”). Therefore, initiation of ECMO support will be required to provide temporary circulatory support and maintain vital organ perfusion as a bridge to definitive therapy. ECMO is a highly advanced modality; however, its ongoing management is technically demanding because it carries multiple risks. ECMO is challenging to maintain because it is associated with multiple risks. These include circuit thrombosis due to clot formation within the extracorporeal circuit and bleeding complications related to systemic anticoagulation administered to prevent thrombosis. ECMO also carries a risk of device-related infection, including infections involving the circuit and cannulation sites. Moreover, ECMO is not a definitive therapy but rather a temporizing, life-sustaining modality; therefore, prolonged use is inherently difficult.

Regarding the anticipated clinical course, there remains a possibility of recovery while on ECMO support, as definitive therapies-such as antimicrobial treatment and, when indicated, surgical intervention-are administered. However, please understand that if antimicrobial therapy is ineffective and surgical intervention is unsuccessful or not feasible, the patient may not survive despite maximal supportive care. At this time, we remain cautiously optimistic and will continue to provide the best possible care using all available resources. We are fully committed to doing everything we can, and we will face this situation together. If you have any questions or concerns, please do not hesitate to ask at any point.

Q2. If the likelihood of clinically meaningful recovery becomes extremely low, you may need to consider discontinuing ECMO support. You also need to discuss in advance whether to attempt cardiopulmonary resuscitation, including chest compressions and defibrillation, if the patient’s heart stops. In some situations, life-sustaining therapies such as ECMO and resuscitation efforts may not benefit the patient and may instead increase suffering. Please communicate these considerations with compassion, answer any questions, and help clarify the patient’s and family’s values and preferences to guide the most appropriate plan of care.

Response 1 (Physician-only group)

As you have closely followed the patient’s clinical course with our team, we believe that you have an overall understanding of the patient’s current clinical status. Although it is difficult to convey, the patient’s overall condition is extremely poor, and the likelihood of meaningful recovery is exceedingly low. In this setting, ECMO is increasingly functioning as life-sustaining therapy rather than a bridge to recovery. In addition, systemic anticoagulation required to maintain ECMO circuit patency may impair hemostasis, resulting in refractory bleeding and subsequent multi-organ failure. Accordingly, continued ECMO support may prolong the current course of intensive care and may inadvertently extend the patient’s suffering by maintaining a burdensome condition without a realistic prospect of recovery. In such circumstances, if circuit exchange becomes necessary due to thrombus formation or other circuit-related complications, one management option would be to **forgo circuit replacement and discontinue ECMO support.**

You may also have heard that, in the event of cardiac arrest, resuscitative measures-such as chest compressions and defibrillation-may be performed. In the present case, given the current severity of cardiac dysfunction, the likelihood that cardiac function would recover sufficiently to sustain effective circulation-even if chest compressions and defibrillation were attempted-is expected to be extremely low. Rather, chest compressions are expected to result primarily in painful iatrogenic harm-such as rib fractures-without meaningful clinical benefit for the patient. Therefore, we would like to discuss with you and establish in advance a clear plan regarding whether to attempt resuscitation in the event of cardiac arrest.

We would appreciate it if you could discuss these two points and share with us your thoughts regarding the plan of care moving forward.

We recognize that we have had to discuss very difficult and distressing matters. We fully understand your hope for even a small possibility of recovery, and as clinicians, we acknowledge and respect those feelings. We understand that you may feel overwhelmed or confused given the amount of information discussed. We are happy to repeat explanations as needed, and we encourage you to ask any questions at any time without hesitation.

Response 2 (AI-only group)

We have continued to provide maximal supportive therapy to date; however, multiple organs—including the heart and lungs—have suffered profound injury, and regrettably, the likelihood of recovery is exceedingly low. ECMO and mechanical ventilation are life-sustaining therapies; however, given the current clinical context, the likelihood of a meaningful functional recovery and return to the patient’s baseline status is exceedingly low.

Additionally, even if the patient were to experience cardiac arrest, attempted resuscitation with chest compressions and defibrillation would impose a substantial physiologic burden, and meaningful recovery would be exceedingly unlikely. Given the patient’s values, wishes, and the kind of life the patient had considered important, it may be most appropriate to prioritize comfort-focused care aimed at minimizing suffering and supporting a peaceful course. Certainly, the final decision will be made through careful, shared deliberation with you.

Response 3 (Physician-AI collaborative group)

I will summarize the patient’s clinical course to date and outline the planned management and anticipated clinical trajectory. What I am about to discuss may be particularly difficult for you to hear. Despite ongoing treatment to date, there has been little to no recovery of cardiac function, and the function of other organs has also shown minimal improvement. Unfortunately, the patient’s prognosis remains extremely poor, and survival is unlikely even with continued maximal medical therapy. I can understand why you would want to hope for a miracle. If we were in your situation, we would likely feel the same way. However, when an infection has led to organ dysfunction of this severity, it becomes extremely difficult to sustain life despite continued intensive treatment.

In the current situation, where meaningful survival is unlikely, continued ECMO support may serve only to prolong the dying process and increase the patient’s burden; therefore, we believe it may be difficult to justify ongoing ECMO. If the ECMO circuit becomes occluded by thrombus and is no longer functional, we are considering treating the current circuit as the final one and not performing further circuit exchanges. In addition, if the ECMO circuit becomes abruptly occluded by thrombus or if emergency discontinuation of ECMO is required, we would proceed with ECMO withdrawal.

Even while ECMO is running, cardiac arrest can still occur. In such circumstances, resuscitative interventions such as chest compressions may cause significant iatrogenic injury, including rib fractures and intraabdominal injury. These measures may therefore serve only to increase the patient’s burden and suffering without meaningfully improving the likelihood of survival with an acceptable quality of life. We aim to prioritize the patient’s comfort by minimizing suffering and enabling the patient to spend this time as peacefully as possible. Accordingly, we believe that the most appropriate plan of care is to forgo further life-prolonging interventions or resuscitative measures that would impose additional burden on the patient, and instead to prioritize comfort-focused care while enabling the patient to spend the remaining time with their family. The final decision will be made together with the family after careful discussion, and we will continue to consider this thoughtfully moving forward.

Q3. The patient’s spouse remarked, “I understand what you are saying, but part of me still hopes for a miracle,” indicating that they had not yet fully relinquished hope. Please speak to the patient’s family clearly and empathetically, taking their feelings into account.

Response 1 (Physician-only group)

We fully understand your wish to hold on to even the slightest hope that there may still be some possibility. Throughout the course of treatment up to this point, we too have faced the patient’s illness each day with the same hope, and for that reason, we deeply understand how you feel.

The decision regarding continuation of ECMO as life-sustaining therapy and the use of resuscitative measures such as chest compressions were made with a full understanding that the possibility of recovery was extremely limited. Nevertheless, it is not difficult to imagine that you may still be experiencing ongoing conflict about whether those decisions were appropriate.

If you feel uncertain or would like further clarification at any time, please do not hesitate to let us know. We will continue to listen to your concerns and provide further explanation whenever needed.

Response 2 (AI-only group)

Hoping for a miracle is a natural emotional response. When it is someone you have loved and lived with for a long time, it is understandable for you to hold on to the possibility that an unexpected recovery may still occur. We, too, have provided treatment while holding on to hope until the very end, and we have no intention of denying or invalidating those feelings now. However, in light of the patient’s current condition, it may be a more compassionate option to allow the patient to spend the remaining time peacefully with you, rather than continuing to receive life-sustaining treatment while enduring ongoing suffering. We will continue to support you and speak with you as we face these uncertainties and concerns together.

Response 3 (Physician-AI collaborative group)

Thank you for openly sharing your honest feelings with us. We understand well your hope for a miracle. That is a completely natural emotional response, and we believe that, if we were in your position, we would likely feel the same way. However, in patients such as this one, in whom infection has caused severe dysfunction of multiple organ systems, sustaining life becomes extremely difficult. At this stage, we should consider together how the patient may spend the remaining time as peacefully as possible. This is an important matter not only for the patient, but also for the family. We will continue to provide our full support and to engage in these discussions together with you.

Q4. The patient’s spouse appeared profoundly fatigued after a prolonged period of bedside attendance and was occasionally tearful while holding the patient’s hand and watching over the patient. The spouse remarked, “Today, my spouse appears to have a slightly better facial expression.” Considering the psychological burden on the patient’s spouse, you spoke in a clear and empathetic manner.

Response 1 (Physician-only group)

Thank you for your continued dedication. It is likely that the patient feels reassured by having you at the bedside. Thank you for your devoted and constant presence at the bedside and for continuing to encourage the patient. Today, the medications being administered to relieve discomfort, including analgesics, may be providing more effective symptom control. As the patient appears somewhat more comfortable today, we would encourage you to leave the patient in our care and take this opportunity to rest as well, without hesitation.

Response 2 (AI-only group)

Thank you very much for remaining at the patient’s bedside each day. The patient’s slightly more relaxed facial expression may reflect a sense of reassurance derived from your presence. We understand that you may be becoming increasingly fatigued after such a prolonged period of bedside attendance. We believe that the patient continues to feel sufficiently reassured simply by your presence, and we would therefore encourage you to take some time to rest, even briefly. We will continue to watch over the patient closely and provide attentive care.

Response 3 (Physician-AI collaborative group)

We sincerely thank you for remaining at the patient’s bedside and watching over the patient each day. From our observations as well, the patient appears to have a better facial expression today than usual, possibly because the analgesic treatment is providing effective symptom relief. The patient has undergone many treatments thus far and has endured them with remarkable strength. We believe you may also be experiencing considerable fatigue, and we would therefore encourage you to take some time to rest. We believe that this is also what the patient would have wished. We will continue to watch over the patient closely and provide attentive care.

Q5. The patient’s spouse stated, “I am truly grateful to all of you. However, I still occasionally question whether the decisions we made were truly the right ones.” You then responded in a clear and empathetic manner.

Response 1 (Physician-only group)

The patient was able to spend a peaceful final period surrounded by family because of your support, cooperation, and the warmth of your care. We, the medical staff, felt that it was a meaningful and peaceful time for both the patient and the family.

We can well imagine that, after having to make important decisions at several turning points during a rapidly changing clinical course over a short period of time, you may still sometimes question whether those decisions were truly the right ones. These feelings may resurface unexpectedly, even now. We understand that it may still be emotionally difficult to reflect on everything from a broader perspective at this time. However, the fact that the patient was able to spend the final moments peacefully is, in itself, evidence that your decisions were not wrong. If there is anything that concerns you, please do not hesitate to ask or speak with us.

Response 2 (AI-only group)

We understand how you feel. When someone you love is involved, it is natural to wonder whether you should have done something differently. But you always tried to do what was best for the patient and did everything possible to reduce the patient’s suffering. Those decisions were not wrong. We believe that the patient would also be grateful. All of us on the medical team sincerely respect the decisions made by you.

Response 3 (Physician-AI collaborative group)

We understand your feelings very well. No matter how carefully such decisions are made, it is entirely natural to feel uncertain and wonder afterward whether they were truly the right ones, especially after losing a loved one. However, because you continued to support the patient and always kept the patient’s best interests in mind, the patient was able to spend the remaining time peacefully. We believe that you made the right decisions. All of us on the medical team are also sincerely grateful that we were able to be with you during that time.

[Scenario2: severe respiratory failure in a patient with malignancy]

Q1. The patient’s spouse, appearing extremely distressed, said to you (the intensivist), “Why has my spouse’s condition suddenly worsened to this extent? Please do anything possible to save them.” In response, you explained the patient’s current condition, the reasons for endotracheal intubation and mechanical ventilation, and the expected clinical course in a clear and empathetic manner.

Response 1 (Physician-only group)

As your attending physician may already have explained, the patient has acute myeloid leukemia, which is a type of blood cancer. We understand that the patient initially visited the previous hospital because of symptoms such as fatigue and shortness of breath. Without treatment, it is a highly aggressive disease with a very poor prognosis, and life expectancy after diagnosis is generally limited to several weeks to a few months.

The patient is currently being treated in collaboration with the hematology team; however, the disease has progressed rapidly, and leukemic involvement has extended to the lungs. As a result, the patient developed severe respiratory distress, and mechanical ventilation was initiated to support breathing. The patient is currently being kept sedated. Although chemotherapy was initiated 3 days ago, the overall condition remains very critical.

Response 2 (AI-only group)

The patient had recently been diagnosed with acute leukemia, a type of blood cancer, and had just begun treatment. However, leukemic cells also infiltrated the lungs, and the patient’s respiratory condition suddenly worsened. At present, the patient is unable to take in enough oxygen through spontaneous breathing alone and is therefore receiving ventilatory support through an endotracheal tube. The condition is extremely critical, and there is a possibility that respiratory distress and overall physical decline may continue to progress. However, the use of mechanical ventilation allows us to continue treatment while helping to reduce the patient’s breathing discomfort as much as possible. We understand that this sudden change must be very distressing for you. We will continue to support both the patient and the family to the best of our ability, and we will remain by your side as we monitor the situation together.

Response 3 (Physician-AI collaborative group)

It is entirely understandable that you are shocked by how suddenly the patient’s condition has worsened. We will explain the patient’s current condition as clearly as possible. The patient has leukemia, a type of blood cancer, and leukemic cells have now spread to the lungs. As a result, the lungs are no longer functioning adequately, making it extremely difficult for the patient to take in enough oxygen. Oxygen administration by mask alone was not sufficient, and the patient’s condition had become life-threatening. Therefore, we initiated ventilatory support with an endotracheal tube to assist breathing. We also want to do everything possible for the patient; however, to speak frankly, the patient’s current physical condition is too poor to tolerate intensive chemotherapy aimed at treating the leukemia itself. Although mechanical ventilation may support the patient’s breathing for a limited period, we must honestly say that the likelihood of long-term improvement is very low. We understand that this is extremely difficult news to hear and may be very hard for you to accept. Even so, we will do everything we can to help the patient remain as comfortable as possible by providing treatment to relieve pain and breathing distress. We will also continue to support you closely throughout this process. Please do not hesitate to speak with us at any time if you have any questions or concerns.

Q2. You explained the patient’s condition since admission to the ICU and the expected clinical course in a clear and empathetic manner to the patient and family.

Response 1 (Physician-only group)

Treatment was initiated on the general ward; however, because the patient developed worsening respiratory failure and severe breathing difficulty, the patient was transferred to the ICU and mechanical ventilation was initiated. Mechanical ventilation is not a definitive treatment for leukemia itself, but rather a supportive measure to relieve respiratory distress.

Regarding the current status of the leukemia, after repeated discussions with the hematology team and ICU staff, we believe that the patient has sustained damage to multiple organs and would be unable to tolerate chemotherapy. Without chemotherapy, it is not possible to halt the progression of the leukemia. Specifically, we believe that the patient’s remaining time may be limited to several days to about one week.

This reflects the limitations of current medical care, and we are deeply sorry that we are unable to do more.

Response 2 (AI-only group)

Even after admission to the ICU, the patient’s respiratory function has continued to gradually decline. Under normal circumstances, chemotherapy would be needed to reduce the burden of the leukemia; however, the patient’s current physical condition is too poor to tolerate such treatment, and it has therefore not been possible to initiate it. In the current situation, it is unlikely that the patient will be able to be weaned from mechanical ventilation, and the possibility of recovery remains extremely low even if treatment is continued. One option is to focus on reducing suffering and avoiding a prolonged period of distress. We will continue to think through these decisions together so that the patient and the family determine the most acceptable course of care.

Response 3 (Physician-AI collaborative group)

Dear family members, I understand that this is an extremely difficult time for all of you, and that you must be feeling exhausted. I would like to take this opportunity to explain the patient’s current condition once again. Since admission to the ICU, the patient’s breathing condition has unfortunately worsened further. The patient is now being fully supported with a mechanical ventilator. Although we would very much like to continue treatment, the patient is currently too physically weakened to tolerate the chemotherapy that would normally be required to treat the leukemia, and administering it may instead place an excessive burden on the body. Therefore, it is extremely difficult in reality to proceed with treatment aimed at curing the disease itself. Although we can continue treatment with mechanical ventilation, I must honestly tell you that there is very little chance that the patient’s condition will improve. Rather, there is a high likelihood that continuing treatment with the patient connected to the ventilator for a prolonged period may only extend the patient’s suffering. From a medical perspective, we believe that the time remaining is likely to be several days to about one week. We understand that having this discussion is extremely painful for all of us, and even more difficult for you to accept. However, our highest priority will be to provide treatment aimed at relieving the patient’s pain and suffering, so that the patient can spend this time as peacefully and comfortably as possible. We will also continue to support you and remain by your side throughout this process. If you have any questions or concerns, please do not hesitate to ask.

Q3. You are now asked to explain the patient’s treatment plan once again to the patient and the family, from both the medical perspective and the patient’s own wishes. While being mindful of the family’s feelings, please provide a clear and empathetic explanation of the future treatment plan, with the greatest possible respect for the patient’s autonomy, including the transition to palliative-focused care.

Response 1 (Physician-only group)

I would like to discuss the future treatment plan. Since definitive treatment for the leukemia is no longer possible, and the patient has expressed a wish to avoid further suffering, our plan is to prioritize the patient’s comfort. We believe that the most appropriate approach is to provide palliative care, using medications such as analgesics to minimize pain and distress during the time remaining. Our goal is to relieve suffering as much as possible and to help the patient spend this time with the family in the most meaningful way possible.

We truly understand how strongly you hope for the patient to recover. If there were any truly effective treatment that we could offer with current medicine, we would very much like to do so. However, no such option is available at present, and we believe that the patient’s suffering and discomfort must now be our highest priority. Therefore, we would like to focus on palliative care.

Response 2 (AI-only group)

The patient clearly expressed a wish not to undergo any further burdensome treatment. We believe that this was not a statement of despair, but an important decision made after carefully facing the patient’s own physical condition. In palliative care, the aim is not to cure the underlying disease, but to alleviate pain, dyspnea, and anxiety as much as possible, while supporting the patient in spending the remaining time in a manner consistent with the patient’s values and dignity. We understand that you naturally wish for the patient to continue fighting for a little longer, and we share those feelings. However, we also believe that helping the patient spend the final stage of life in accordance with their wishes is an equally important expression of love and support.

Response 3 (Physician-AI collaborative group)

We sincerely understand your feelings. It is only natural for you to wish to hold on to hope, to want the patient to recover again, and to want to do everything possible to help. We too sincerely wish that the patient could return to the previous condition, if that were possible. However, from a medical standpoint, the patient’s current condition no longer permits curative treatment for the underlying disease. Unfortunately, even if mechanical ventilation is continued, it is likely to increase the patient’s suffering and burden rather than improve the condition. At the same time, the patient had previously expressed a clear wish not to undergo any further burdensome treatment and to remain as free from suffering as possible at the end of life. Above all, we believe that the patient’s own values and wishes should be respected as the highest priority. Therefore, rather than pursuing further aggressive disease-directed treatment, we would like to propose a transition to palliative care focused on alleviating dyspnea, pain, and anxiety, while supporting the patient in spending the remaining time as peacefully and authentically as possible. This should not be understood as giving up. Rather, it means that we will do everything we can to help the patient spend the remaining time as peacefully as possible, together with you, in a manner consistent with the patient’s values and identity. We would like to move forward together with you, carefully considering how best to support the patient during this important time and what would be most beneficial for the patient. Please do not hesitate to share any questions or concerns you may have.

Q4. When responding to the patient’s questions, please offer supportive and empathetic words in a clear and understandable manner, while being attentive to the patient’s feelings of guilt and emotional conflict.

Response 1 (Physician-only group)

Because you had been well until recently, we understand that suddenly hearing about a limited prognosis can be profoundly overwhelming. Although I am speaking to you now as a physician, I believe that if I or a member of my own family were placed in a similar situation, I would likely feel the same way, even with medical knowledge. For individuals without a healthcare background, such feelings may be even more understandable. We hope to discuss the future course of care carefully and thoughtfully, so that you and your family can move forward without regret; however, it is necessary to clarify what should be prioritized above all else. Whether priority should be given to your suffering or to your family’s wishes is not a matter of right or wrong. However, it is you who is directly experiencing the physical pain and distress. Many patients in similar situations feel that they should continue enduring treatment because they do not want to let their families down. In my personal view, the most important priority is to minimize your suffering, and this is the perspective I usually share with your family in such circumstances. In many cases, the more family members meet with a patient who is struggling with serious illness, the more they express a wish for the patient to remain free from suffering. When your family arrives later, I will explain what we have discussed today.

Response 2 (AI-only group)

It is entirely natural to feel conflicted between wanting to live up to your family’s hopes and wanting to remain true to your own feelings. Expressing that you can no longer continue is by no means a betrayal of your family. Rather, honestly expressing your wishes is also important for your family. We will do our utmost to support you so that you can spend this time with peace and reassurance. We will also help ensure that your thoughts and wishes are clearly communicated to your family.

Response 3 (Physician-AI collaborative group)

There is no need to blame yourself in that way. It is truly alright. When your family says, “Keep fighting” or “Don’t give up,” it is because they love you deeply and care about you very much. However, perseverance can take many different forms. You have already lived a life of dedication, serving society as a police officer with a strong sense of justice. You have also built many precious memories with your family over the years. That alone reflects more than enough strength and perseverance. At this stage, choosing to focus on relief from pain and suffering and to spend the remaining time peacefully, rather than continuing burdensome treatment, is also a meaningful and courageous form of perseverance. This should not be understood as either a betrayal of your family or an act of giving up. Rather, it is likely that your family’s deepest wish is for you to remain free from suffering and to spend this time peacefully. I also believe that this is the kindest choice for you. Both your wishes and your family’s love are profoundly important. Please do not blame yourself, and I hope that the remaining time can be spent meaningfully and peacefully together with your family.

Q5. At a later time, the family member said, “Thank you very much for everything you have done for us. At first, I was unable to accept the reality of the situation, and I found it difficult to accept their wishes to discontinue treatment. I even said to them, ‘Keep fighting’.” Please speak to the patient’s family in a way that is both clear and empathetic.

Response 1 (Physician-only group)

Very few people are able to remain calm when a loved one who had previously been well suddenly becomes critically ill and they are told that the expected prognosis is only a few weeks. As a healthcare professional, I have cared for many families in similar situations, and many have shared similar feelings.

The time remaining is likely to be limited. If there is anyone you would like the patient to see, we would encourage you to invite them to visit. We also hope that you will be able to spend this time together in a way that leaves as few regrets as possible. However, I do not believe that the patient would want your health to deteriorate from staying at the hospital without rest. Please go home as needed and take time to rest. If there is any worsening in the patient’s condition, we will contact you immediately.

All of our staff will make every effort to keep the patient as comfortable as possible. If you have any concerns or questions, please do not hesitate to tell us.

Response 2 (AI-only group)

I fully understand that, at first, it was difficult for you to accept the situation, and that you encouraged your loved one to “keep fighting” because you hoped the patient might live even a little longer. However, your eventual decision to respect the patient’s wishes was an expression of profound love. I believe that the patient would also be grateful for the support you provided until the very end. Our medical team deeply respects those feelings and holds them with great care.

Response 3 (Physician-AI collaborative group)

Thank you for sharing your feelings with us. By speaking so openly, you help us better understand what you are going through. When your spouse, someone very dear to you, was placed in such a situation, it was entirely understandable that you wanted to say, “Let’s keep fighting” or “Please don’t give up.” Wanting your loved one to recover somehow and not wanting to lose them were natural feelings, and there was nothing wrong with having those thoughts. Rather, those words were an expression of your deep love for them. At the same time, it was truly meaningful that the patient was able to clearly communicate those wishes to us, and above all, that you were able to accept them and think through these difficult decisions together with the patient. Although it was initially difficult for you to accept the situation, you chose to respect the patient’s wishes and remain close to the patient. I believe that, because of this love and support, the patient was able to spend the final days peacefully, surrounded by beloved family members. I believe that the patient felt your deep love and was now resting peacefully, free from suffering. Please do not blame yourself. Rather, I hope you will take pride in the deep love and support you showed the patient by remaining close to the patient until the very end.

[Scenario3: post-cardiac arrest syndrome]

Q1. The patient’s son or daughter, with a look of mixed anxiety and hope, asked you (the intensivist), “Since the heart has started beating again, does that mean they will be all right? Will they recover and become well again?” Please provide a clear and empathetic explanation of the patient’s current condition, the treatment options to be considered from this point forward, including targeted temperature management, and the expected clinical course.

Response 1 (Physician-only group)

The patient suddenly collapsed at a supermarket, and an ambulance was called. A bystander immediately initiated chest compressions, and after the arrival of emergency medical services, return of spontaneous circulation was achieved with a single defibrillation shock. However, the patient remained unconscious and was transported to the hospital. Although chest compressions were initiated promptly, the patient remained in cardiac arrest for approximately ten minutes. Computed tomography (CT) showed mild findings suggestive of hypoxic brain injury sustained during that period. The patient is currently intubated and sedated to support breathing. No major abnormalities in cardiac function have been identified at this time.

To be honest, at this point, we cannot say with certainty whether the patient will regain consciousness. Over the next few days, we would like to initiate targeted temperature management, a treatment that lowers the body temperature in an effort to reduce further brain injury. Targeted temperature management will be continued for 3 days, after which sedation will be reduced to evaluate whether the patient regains consciousness. We understand that this situation has developed suddenly and may be overwhelming for you. We also recognize that the many uncertainties regarding the patient’s future course may be a source of significant concern. Please feel free to ask any questions if there is anything you were unable to ask earlier or if anything is causing you concern. At this point, we believe that the most important step is to proceed carefully with targeted temperature management in order to reduce the extent of brain injury caused by the cardiac arrest.

Response 2 (AI-only group)

The patient’s heart has indeed started beating again. However, there was a period after the collapse during which the brain did not receive enough oxygen, and as a result, the brain has developed swelling. The brain is extremely sensitive, and even a few minutes without adequate oxygen can lead to serious and lasting damage. We will now begin targeted temperature management, a treatment that temporarily lowers the body temperature. The purpose of this therapy is to reduce ongoing brain injury as much as possible by lowering the body’s temperature.

Even with this treatment, there remains a possibility that the patient may not regain consciousness. However, we will do everything we can. We understand that this is an extremely distressing and uncertain time for you, and we will continue to support you in every way possible.

Response 3 (Physician-AI collaborative group)

I understand that this has happened very suddenly and must be extremely upsetting. I would like to explain the patient’s current condition, the treatment we are going to provide, and the expected course as clearly as possible. The patient suddenly collapsed because the heart stopped beating, but it started beating again after cardiopulmonary resuscitation and electrical defibrillation. The fact that the heart, once stopped, has started beating again is certainly a sign that the patient is alive. However, while the heart was not beating, the brain may not have received enough blood flow and may have been injured because of a lack of oxygen. At present, the patient is not responding to verbal stimulation and remains unconscious, which may reflect severe injury to the brain. In addition, the cause of the cardiac arrest has not yet been identified at this time, but we will continue to investigate it carefully.

We will begin a treatment called targeted temperature management, in which the body temperature is lowered slightly to help prevent further brain injury. By keeping the body temperature from rising, this treatment aims to reduce inflammation and swelling in the brain and to prevent additional damage to the brain. The effects of this treatment are not seen immediately. We will carefully control the body temperature over the next several days and then reassess the condition of the brain. Regarding the prognosis, it is still too early to determine how much the brain injury may progress, how much it can be reduced, or whether the patient will eventually regain consciousness. We understand your concern. At this stage, our focus is on providing all possible treatment while closely observing the patient’s condition. We also recognize how difficult and uncertain this situation must be for you, and we encourage you to contact us at any time if there is anything you would like to ask or discuss.

Q2. You are asked to explain to the patient’s family, in a clear and empathetic manner, the patient’s condition after targeted temperature management, the examination findings, and the expected clinical course.

Response 1 (Physician-only group)

After targeted temperature management, we performed a follow-up CT scan. Unfortunately, the interruption of blood flow to the brain during the period of cardiac arrest appears to have caused substantial damage, and the brain injury was more severe than we had initially expected. After careful discussion among the ICU team, the neurology specialists, and the staff involved in the patient’s care, we concluded that the possibility of the patient regaining consciousness is low.

Regarding the future course, the patient’s heart is currently beating steadily, but there remains a possibility of sudden deterioration. If there are family members who may want to see the patient, we would recommend informing them. Going forward, the patient may need a tracheostomy, which is a procedure to change from the breathing tube in the mouth to one inserted through the neck, and nutritional support through a feeding tube may also become necessary. If the patient’s condition becomes more stable, transfer to a long-term care facility may be possible. However, I am sorry that we cannot be more definite about this at the present time.

Since the patient collapsed, chest compressions have been performed and many emergency medications have been given. A tracheostomy would also require surgery. If the patient had ever shared thoughts with you about what they would want in a situation like this, we believe it is important to respect those wishes as much as possible. In any case, rather than putting the patient through further invasive procedures or additional burden, we believe that focusing on a more natural course may also be an appropriate option. If the patient’s condition changes, we can continue to discuss the treatment plan together at each stage.

Response 2 (AI-only group)

Although targeted temperature management was performed, the patient has unfortunately not regained consciousness. Further detailed evaluation showed that the brain has suffered severe damage. From a medical perspective, the possibility of the patient regaining consciousness from this condition is considered to be extremely low.

We understand that the patient had previously said that, if survival was no longer possible or if consciousness was lost, the patient would wish to be allowed a natural course. Respecting those wishes is a very important choice. At the same time, there is no need to come to a decision right away, and we can continue to talk this through carefully together with you.

Response 3 (Physician-AI collaborative group)

I would like to explain again the patient’s current condition and the expected course. Although we performed targeted temperature management for 3 days, the patient has unfortunately not regained consciousness. The head CT and electroencephalography showed that the brain has suffered severe damage. Based on these findings, the possibility of the patient regaining consciousness is considered to be extremely low.

Of course, we cannot say that the possibility is completely zero. However, in reality, the hope for recovery is extremely small. We understand how painful it is to hear this so suddenly, and we would like to continue considering the next steps together with you, while carefully respecting the wishes and feelings of both the patient and the family.

Q3. The spouse and children have differing views and have not yet been able to decide on the treatment plan. You are asked to explain the future course of treatment to the family in a clear and empathetic manner, while respecting the family’s feelings and addressing both the patient’s wishes and the medical perspective.

Response 1 (Physician-only group)

Based on the CT findings, the possibility of the patient regaining consciousness is considered to be extremely low. The brain is very vulnerable, and even a few minutes without circulation can cause serious injury. Although targeted temperature management was provided, the damage and swelling in the brain were unfortunately extensive, and the chance of the patient regaining consciousness is thought to be almost zero.

Since the patient collapsed, we have done everything we could medically, including electrical defibrillation, chest compressions, and targeted temperature management. At the same time, these treatments have also placed a significant burden on the patient’s body. As you have expressed, we believe that the patient has already fought very hard. The heart, which had once stopped, began beating again, and the patient was able to arrive at the hospital. However, if the heart were to stop again in the future, further chest compressions could cause injuries such as rib or sternal fractures. There are also advanced treatments, such as the use of extracorporeal cardiopulmonary support, but these would place an even greater burden on the patient. As you have shared that the patient had previously discussed these situations with you, it may be appropriate to respect the patient’s wishes. This may mean allowing the course to proceed naturally and supporting the patient in spending the final stage of life peacefully, surrounded by loved ones.

We understand that this has all happened very suddenly, and it may be hard for you to take everything in right away. Fortunately, the patient’s heart is stable at this time, and because this is a very important matter, it may be helpful for you to take some time, talk it over together as a family, and think about it further at home. When you have come to a decision, please let us know.

Response 2 (AI-only group)

We understand your feelings very well. As a family, it is entirely natural to think, “If the possibility is not absolutely zero, we will want to hope.” At the same time, the patient had previously expressed a wish to allow the course to proceed naturally. Medically, the possibility of the patient regaining consciousness is very low, and further treatment would likely place a substantial burden on the body and prolong suffering. Taking into account both the patient’s wishes and the current medical circumstances, we believe that shifting the focus to palliative care, aimed at easing suffering and helping the patient spend this time as peacefully and naturally as possible, may be the kindest choice for both the patient and the family. We would like to continue working together with you to find the best possible way forward for the patient.

Response 3 (Physician-AI collaborative group)

It is very natural for family members to have different feelings in a situation like this. It is completely understandable to hope that, if there is any possibility at all, treatment should continue. At the same time, it is also understandable for you to feel that treatments the patient would not have wanted should not be prolonged. Both of these feelings reflect how deeply you care about the patient.

However, from a medical perspective, the possibility that the current degree of brain injury will improve dramatically and allow the patient to regain consciousness is extremely low. We also understand that the patient had previously expressed a wish to be allowed a natural course if consciousness was lost. We believe that the patient’s own wishes should be given the highest priority. From this point forward, we believe that focusing on palliative care, with the aim of reducing suffering and discomfort as much as possible and allowing the patient to remain as peaceful as possible, would be the approach most consistent with the patient’s wishes.

We would not want this treatment plan to be misunderstood as something that goes against the wishes of the patient, the spouse, or the children. Our aim is to support the patient in spending this time in a way that remains true to the patient’s value and identity, and we will do everything we can to help. If you have any concerns or questions, please do not hesitate to share them with us at any time.

Q4. You are now going to explain to the patient’s family why a tracheostomy is necessary. Please explain, in a clear and empathetic manner, the reasons for performing a tracheostomy in the current situation, as well as its advantages and disadvantages.

Response 1 (Physician-only group)

One advantage of tracheostomy is that it allows more stable respiratory management than an endotracheal tube placed through the mouth. It may also increase the possibility of weaning the patient from mechanical ventilation. In addition, airway secretions can be suctioned more easily, and oral care becomes easier to perform, which may help prevent pneumonia. Another advantage is that the area around the mouth appears less crowded and easier to manage. On the other hand, because this is a surgical procedure, it carries important risks, including bleeding and infection, and therefore must be performed with great caution. At this stage, the patient’s condition appears to be becoming more stable, and a tracheostomy may therefore be an appropriate option for ongoing management. If this procedure is performed, we will make every effort to minimize the burden on the patient as much as possible.

Response 2 (AI-only group)

At present, the patient is breathing through a tube placed through the mouth. However, if this tube is used for a prolonged period, there is a risk of injury or infection involving the mouth and throat. A tracheostomy is a procedure in which a tube is inserted directly through the front of the neck, which can reduce the burden on the mouth and throat. In addition, if the patient’s breathing becomes more stable, it may be possible to remove the ventilator and continue management with the tracheostomy tube alone.

However, because recovery of consciousness is not expected, this procedure would be performed not so much to prolong life as to prevent complications and help the patient remain as comfortable as possible. We would like to make this decision together with you while carefully respecting your feelings.

Response 3 (Physician-AI collaborative group)

As for the patient’s current condition, consciousness has not yet returned, but the blood pressure and oxygen levels are stable. At present, breathing is being supported with a tube placed through the mouth. However, if this tube remains in place for a prolonged period, it can cause injury to the mouth and throat and increase the risk of infection. The patient still requires ventilatory support, so the tube placed through the mouth cannot be removed at this time. For this reason, we would like to propose switching to a tracheostomy, a procedure in which a breathing tube is inserted directly through the front of the neck.

The benefit is that, once the tube is removed from the mouth, it becomes easier to keep the mouth clean, and this may reduce the patient’s discomfort. On the other hand, this requires a surgical procedure to make an opening in the neck for the tube, so a scar will remain and there is a risk of infection at the procedure site. However, for patients who need long-term ventilator support, this procedure is often necessary. We believe that a tracheostomy would be the better option for the patient’s safety and comfort. If you have any questions or concerns, please do not hesitate to ask us at any time.

Q5. The patient’s family is deeply distressed by the possibility that the patient may not regain consciousness and feels uncertain about how to face and support the patient going forward. Please explain, in a clear and empathetic manner, how the family may continue to support the patient and remain by their side while acknowledging their grief and anxiety.

Response 1 (Physician-only group)

Because the patient had been well until recently, it is entirely understandable that you feel deeply confused and distressed by this sudden loss of consciousness and lack of responsiveness. Although the patient remains unconscious, the respiratory and cardiac conditions are stable, which means that important bodily functions are being maintained. It is also possible that hearing may still be preserved, so it may be meaningful for you to touch the patient gently and speak to them.

Regarding future visits, we would encourage you to come in a way that does not place too great a burden on yourselves. We will continue to provide the fullest possible medical support here. If anything is needed or if there is any change in the patient’s condition, we will let you know as soon as possible. We understand that this is a very difficult and worrying time, but please also try to rest, get enough sleep, and take care of your own health.

Response 2 (AI-only group)

Even if consciousness does not return, being at the patient’s side, speaking to the patient, and holding the patient’s hands are by no means meaningless. Research suggests that, even when a patient appears to be unconscious, sounds and the sensation of touch may still be perceived. Above all, the greatest source of reassurance for the patient may be the presence of loved ones nearby.

Going forward, we hope to continue watching over the patient together with you, paying careful attention to even small daily changes and cherishing this time. We will continue to support you in every way we can.

Response 3 (Physician-AI collaborative group)

Thank you for speaking so openly about how you feel. It is very natural to feel anxious about the future. However, even if the patient remains unconscious and does not respond, it is still quite possible that voices, sounds, and touch may be perceived. Even without a clear response, speaking gently to the patient and holding the patient’s hands may help provide a sense of comfort and security.

For you too, this can be a meaningful way of showing, “We are here with you.” As you continue to spend time with the patient, creating a calm and reassuring environment may be one of the most important ways to support them. We, as the medical team, will continue to support you in this process, so please do not feel that you must carry this burden alone. If there is anything at all, we encourage you to speak with us at any time.
