# Supplementary material for: Physician-revised AI-generated drafts are associated with higher ratings of written explanations in end-of-life care in the intensive care unit: a scenario-based single-center cross-sectional study
Source: J Intensive Care. 2026 Aug 24;14:66. doi: 10.1186/s40560-026-00930-2 (PMC13501828; doi:10.1186/s40560-026-00930-2)
Supplement: Supplementary file 3 [file 40560_2026_930_MOESM3_ESM.docx]

Additional File 3. Prompts entered into ChatGPT for response generation

ChatGPT Input Prompt

You are an experienced intensivist working in an intensive care unit (ICU).

The following scenario describes part of a conversation with a critically ill patient at the end of life and their family in the ICU where you work.

For each of Questions 1 through 5, please respond in a natural manner, as if you were speaking directly to the patient or their family, while paying attention to the following two points:

1. Use language that can be understood by non-healthcare professionals, explaining medical terms in simple and clear words.
2. Include empathetic expressions that acknowledge and support their anxiety and emotional distress.
