# Supplementary material for: Physician-revised AI-generated drafts are associated with higher ratings of written explanations in end-of-life care in the intensive care unit: a scenario-based single-center cross-sectional study
Source: J Intensive Care. 2026 Aug 24;14:66. doi: 10.1186/s40560-026-00930-2 (PMC13501828; doi:10.1186/s40560-026-00930-2)
Supplement: Supplementary file 4 [file 40560_2026_930_MOESM4_ESM.docx]

Additional File 4. Inter-rater reliability for the three pre-specified primary outcomes

| **Table S1.** Inter-rater reliability for the three pre-specified primary outcomes | | | |
| --- | --- | --- | --- |
| **Outcome** | **Number of raters** | **Number of response texts** | **ICC (95% CI)^a^** |
| Medical validity score (HCP) | 30 | 45 | 0.116 (0.073-0.186) |
| Empathy score (HCP) | 30 | 45 | 0.283 (0.203-0.397) |
| Empathy score (non-HCP) | 15 | 45 | 0.282 (0.195-0.403) |
| HCP, healthcare professionals; non-HCP, non-healthcare professionals; ICC, intraclass correlation coefficient; CI, confidence interval | | | |
| ^a^ Single-measure ICCs and 95% confidence intervals were calculated across the 45 response texts using a two-way random-effects model for absolute agreement. | | | |
