# Supplementary material for: Physician-revised AI-generated drafts are associated with higher ratings of written explanations in end-of-life care in the intensive care unit: a scenario-based single-center cross-sectional study
Source: J Intensive Care. 2026 Aug 24;14:66. doi: 10.1186/s40560-026-00930-2 (PMC13501828; doi:10.1186/s40560-026-00930-2)
Supplement: Supplementary file 5 [file 40560_2026_930_MOESM5_ESM.docx]

Additional File 5. Comparison of response length across the three response groups

| **Table S2.** Response length across the three response groups | |
| --- | --- |
| **Response group** | **Character count, median (IQR)^a^** |
| physician-only | 336 (259-531) |
| AI-only | 234 (181-248) |
| physician-AI collaborative | 499 (283-645) |
| AI, artificial intelligence; IQR, interquartile range | |
| a Response length was defined as the number of Japanese characters in each final response, excluding spaces and line breaks. | |

| **Table S3.** Overall and pairwise comparisons of response length across the three response groups | |
| --- | --- |
| **Comparison** | ***P* value^a^** |
| Overall difference among the three response groups | **<0.001** |
| physician-AI collaborative vs physician-only | >0.999 |
| physician-AI collaborative vs AI-only | **<0.001** |
| physician-only vs AI-only | **<0.001** |
| AI, artificial intelligence | |
| ^a^ The overall comparison was performed using the Friedman test. Pairwise comparisons were performed using Dunn's multiple comparison test following a significant overall test. A two-sided P value < 0.05 was considered statistically significant. | |
