# Supplementary material for: Physician-revised AI-generated drafts are associated with higher ratings of written explanations in end-of-life care in the intensive care unit: a scenario-based single-center cross-sectional study
Source: J Intensive Care. 2026 Aug 24;14:66. doi: 10.1186/s40560-026-00930-2 (PMC13501828; doi:10.1186/s40560-026-00930-2)
Supplement: Supplementary file 6 [file 40560_2026_930_MOESM6_ESM.docx]

Additional File 6. Response-length-adjusted sensitivity analyses

| **Table S4.** Response-length-adjusted sensitivity analyses for the three pre-specified primary outcomes | | | |
| --- | --- | --- | --- |
| **Outcome** | **Comparison** | **Estimate (95% CI)^a^** | ***P* value^b^** |
| Medical validity score (HCP) | physician-AI collaborative vs physician-only | 0.421 (0.333 to 0.510) | **<0.001** |
|  | AI-only vs physician-only | ‐0.166 (‐0.254 to -0.077) | **<0.001** |
|  | Response length, per 100 Japanese characters | 0.014 (‐0.005 to 0.033) | 0.151 |
| Empathy score (HCP) | physician-AI collaborative vs physician-only | 0.975 (0.854 to 1.096) | **<0.001** |
|  | AI-only vs physician-only | 0.115 (‐0.006 to 0.236) | 0.063 |
|  | Response length, per 100 Japanese characters | ‐0.012 (‐0.042 to 0.018) | 0.426 |
| Empathy score (non-HCP) | physician-AI collaborative vs physician-only | 1.223 (1.041 to 1.405) | **<0.001** |
|  | AI-only vs physician-only | 0.232 (0.050 to 0.413) | **0.013** |
|  | Response length, per 100 Japanese characters | 0.024 (‐0.021 to 0.068) | 0.296 |
| CI, confidence interval; HCP, healthcare professionals; non-HCP, non-healthcare professionals; AI, artificial intelligence | | | |
| ^a^ Estimates and 95% confidence intervals were obtained from linear mixed-effects models including response group and mean-centered response length, with random intercepts for participant and question. Group estimates represent adjusted differences from the physician-only group, whereas the response-length estimate represents the change in the outcome score per 100-character increase. | | | |
| ^b^ *P* values were obtained from the linear mixed-effects models. | | | |
